# Long-Lasting WNT-TCF Response Blocking and Epigenetic Modifying Activities of Withanolide F in Human Cancer Cells

**DOI:** 10.1371/journal.pone.0168170

**Published:** 2016-12-14

**Authors:** Chandan Seth, Christophe Mas, Arwen Conod, Jens Mueller, Karsten Siems, Monika Kuciak, Isabel Borges, Ariel Ruiz i Altaba

**Affiliations:** 1 Department of Medical Genetics and Development, CMU, 1 rue Michel Servet, Geneva, Switzerland; 2 Analyticon Discovery, Biotech Campus Potsdam-Hermannswerder, Potsdam, Germany; University of Kansas, UNITED STATES

## Abstract

The WNT-TCF signaling pathway participates in adult tissue homeostasis and repair, and is hyperactive in a number of human diseases including cancers of the colon. Whereas to date there are no antagonists approved for patient use, a potential problem for their sustained use is the blockade of WNT signaling in healthy tissues, thus provoking potentially serious co-lateral damage. Here we have screened a library of plant and microorganism small molecules for novel WNT signaling antagonists and describe withanolide F as a potent WNT-TCF response blocker. This steroidal lactone inhibits TCF-dependent colon cancer xenograft growth and mimics the effects of genetic blockade of TCF and of ivermectin, a previously reported WNT-TCF blocker. However, withanolide F is unique in that it imposes a long-lasting repression of tumor growth, WNT-TCF targets and cancer stem cell clonogenicity after drug treatment. These findings are paralleled by its modulation of chromatin regulators and its alteration of overall H3K4me1 levels. Our results open up the possibility to permanently repress essential signaling responses in cancer cells through limited treatments with small molecules.

## Introduction

Constitutive activation of the canonical WNT-TCF pathway is a common driver of a number of human cancers. This often occurs through the loss of APC function, allowing ßCATENIN to enter the nucleus, associate with TCF factors and regulate WNT-TCF targets. Several small molecule antagonists of WNT-TCF signaling are in development but so far none are approved for patient use (e.g., [1]).

Given that many tumors harbor an activated pathway downstream of APC, small molecule antagonists that block WNT pathway activity upstream at the level of ligand secretion or signal transduction [2–4] may not be universally useful. Repositioning approved compounds as WNT blockers that affect downstream steps is an exciting possibility [5,6], but the need to maintain drug presence for sustained activity for all known WNT-TCF blockers to date may raise the concern of collateral damage on healthy tissues that require WNT signaling.

To uncover novel WNT-TCF response antagonists acting downstream of APC, we have screened a library of highly diverse plant and microorganism natural compounds with a TCF reporter assay [7] modified to track the activity of repressed TCF [6]. We find that our lead, withanolide F, harbors excellent WNT-TCF repressive activities in vitro and in vivo. Moreover, it is unique in that it induces the long-lasting repression of WNT-TCF targets and of cancer stem cell clonogenicity, modulating the expression of chromatin remodeling components. Our present data uncover the exciting possibility to permanently repress WNT-TCF responses in human cancer cells with natural compounds that alter the epigenetic landscape.

## Materials and methods

### Animal care and statistics

The study used human cells previously used in published research papers. All were obtained and used following approved guidelines of the University of Geneva. All animal procedures were under approved protocols of the Office Cantonal Vétérinaire de Genève. Mice were kept in ventilated cages in a modern facility and monitored several times weekly for well-being under veterinarian guidance. They were euthanized at the end of the experiments, and before tumors reached the local legal limit (15mm in diameter), through injection of ketazol/xylazine or CO_2_ inhalation. All p values are from two-tailed student t-tests using triplicates or as noted.

### Cells, libraries, screens and small molecules

Primary colon cancer cells CC14, CC36 and mCC11 [8], colon cancer DLD1, HT29, Ls174T, melanoma MeWo, glioblastoma U87 and U251, and lung cancer H358 cell lines (ATCC), as well as 293T cells, were cultured in DMEM-F12, 10% FBS.

3076 natural compound and 2468 synthetic analogue (Analyticon Discovery) stocks in 96-well plates were at 10mM after addition of DMSO. Four independent batches of CAP2 (Analyticon Discovery), Ivermectin (Sigma), selamectin (Sigma, Zoetis) and other cardenolides and withanolides (Sigma) were also dissolved in DMSO, with the exception of digoxin, which was dissolved in methanol.

Primary and secondary screens [6] used 293T cells transfected with TOP Firefly and Renilla luciferase plasmids in 96 well plates. Cells were treated with compounds for 16h, washed and lysed. Readouts used the Promega Dual Reporter luciferase kit.

Aerial parts of *Withania adpressa* were collected by Sahara Exporters sarl, (Errachidia, Morocco). Dried plant material was extracted with MTB-ether–methanol. Withanolide F (CAP2) was isolated by repeated reverse phase chromatography. The structure of Withanolide F was confirmed by comparison of NMR and MS data with data reported in the literature [9].

### BrdU incorporation, live imaging and activated Caspase 3 assays

Cells treated in reduced serum (2.5%FBS) were given BrdU (10mg/ml, Sigma) for 20min, washed and fixed with fresh PFA (4%, pH8) followed by acid treatment, neutralization and incubations with anti-BrdU (University of Iowa Hybridoma Bank), and rhodamine-coupled anti-mouse secondary antibodies (Invitrogen Molecular Probes). Nuclei were counterstained with DAPI (Sigma).

Live imaging fluorescence intensity changes (relative fluorescence) of CC14-GFP^+^ cells in microwells were scored using a Widefield plate reader, (ImageXpress XL with an inbuilt temperature and CO_2_ controller, and a CoolSnap HQ camera; Photometrics). Cells were treated for 6hrs and imaged. Active (cleaved) Caspase-3^+^ apoptosis was determined using the BD Pharmigen kit and FACS.

### PCR and DNA constructs

Rt-qPCR were performed [6] using BioRad equipment and reagents. Cells were treated for 16hrs, unless otherwise noted, and collected in Trizol (Ambion) before total RNA extraction and cDNA synthesis. For spheroid analyses GenElute (Sigma) columns were used. Primers were as in S1 Table. *HPRT*, *ßACTIN*, *HMBS* and *TBP* were used as controls for normalization. All heat maps represent unitless ratios of experimental over control mRNA levels, normalizing experimental gene expression with the levels of expression of housekeeping genes. N’Δ ßCATENIN and dnTCF constructs were used as in [6,10].

### Clonogenic spheroid assays

Cells treated for 16hrs in 2D adherent culture were washed, harvested and plated in 3D suspension without drug in serum-free DMEM-F12 media with B27 supplement (1:50 dilution), at 1 cell/well in 96-well format in triplicates. Plated cells were monitored to ensure the presence of a single cell per well and spheroids counted and photographed at day 10.

### Epistatic rescue experiments

72h after transfection of vectors expressing a TCF4^VP16^ and GFP, or vector alone, plus reporter plasmids, cells were visually checked for expression of GFP. Independent batches of cells with >80% GFP expression were chosen and treated in DMEM-F12 (with 2.5% FBS) for 16hrs.

### Microarrays

Quality and quantity of RNA purified with Trizol was checked using Nano Series (Agilent Technologies). Samples with RNA Integrity Numbers (RIN) >9/10 were chosen, prepared using GeneChip IVT Express kit and hybridized to PrimeView U219 human array (Affymetrix) in the Genomic Platform at the Faculty of Medicine, University of Geneva. Data can be accessed at Gene Expression Omnibus (GEO) repository under accession number GSE87875.

### Nude mice xenografts and treatment

0.5x10^6^ DLD1 cells were injected per flank into 6 week-old female Nude mice (Janvier Labs). CAP2 (10mg/kg) complexed with ßcyclodextrin as vehicle, or vehicle alone, were injected every second day intraperitoneally once the tumors were palpable (~5-10mm^3^). Tumor volumes were measured every other day with a caliper. Animals were sacrificed at the end of the experiment and tumors harvested. All animal procedures were under approved protocols of the Office Cantonal de Veterinaire de Genève. CAP2 from four independent batches (Analyticon Discovery) was dissolved in DMSO and suspended in 45% ßcyclodextrin. The latter was used alone for controls.

### HISTONE 3 assays

EpiQuick^TM^ HISTONE 3 modification colorimetric assays (Epigentek) were used to quantify the levels of overall HISTONE 3 modifications in DLD1 cells treated with 5μM CAP2 in DMSO, or DMSO only as control for 16h. Results were read on a Victor screening robot and normalized over total H3 levels as indicated by the manufacturer.

## Results

### Identification of natural steroidal lactones as WNT-TCF signaling response inhibitors

**Fig 1:**
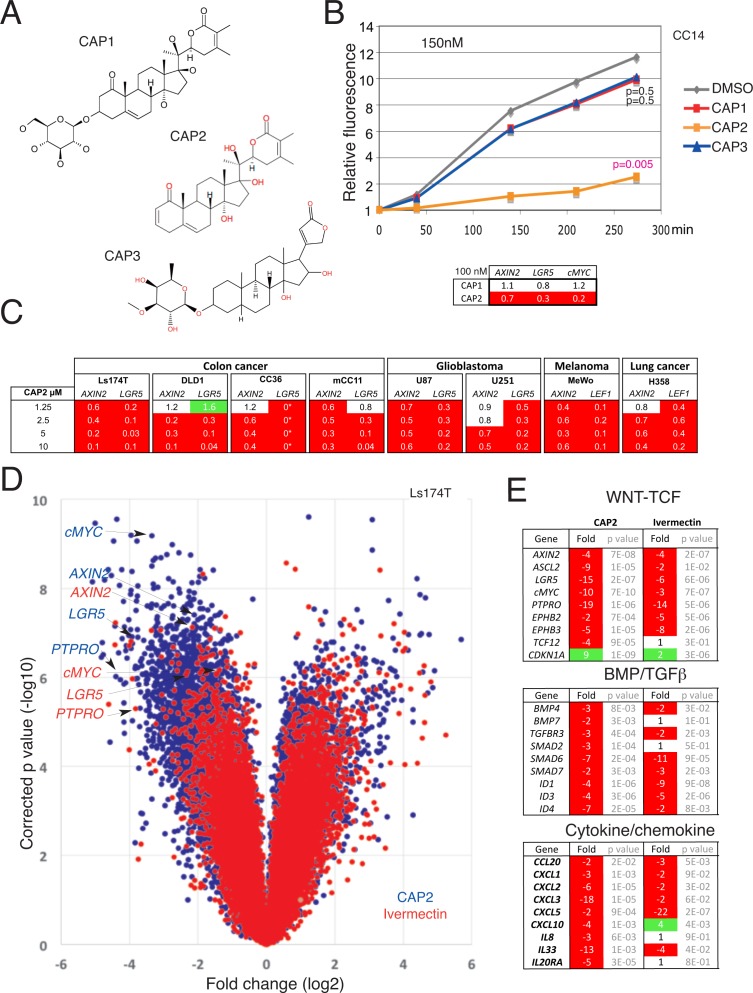
Identification of CAP molecules as WNT-TCF response blockers. A) Chemical structure of the CAP1, CAP2 and CAP3 steroidal lactone small molecules. B) Changes in relative fluorescence intensity of CC14-GFP^+^ cells over time. At 0.15μM only CAP2 is effective whereas at 2.5μM CAP1, CAP2 and CAP3 repress increases in fluorescence (see also panel C in S3 Fig), related here to cell state and mass. P values are given for the end points in relation to DMSO-treated controls. The inset shows a heat map with normalized ratios over control highlighting the repression of *AXIN2*, *LGR5* and *cMYC* by CAP2 but not by CAP1 at 0.1μM. C) Heat maps of the normalized expression levels of different WNT-TCF targets after treatment of multiple colon cancer cells, melanoma, glioblastoma and lung cancer cells with increasing concentrations of CAP2 as noted. *LGR5* levels in CAP2-treated CC36 were undetectable (0*). All values are normalized ratios with DMSO-controls, which were equated to 1. All analyses shown in this figure were performed with the third CAP2 batch. D) Scatter (volcano) plot of the changes in gene expression obtained by microarray of Ls174T cells treated with 5μM CAP2 (blue) or 5μM with Ivermectin (red) for 16h in vitro as 2D cultures. Values are over controls and are per probe with the x-axis giving the fold change in log2 scale and the y-axis the corrected p value in log10. The position of four WNT-TCF targets is highlighted. E) Analyses of WNT-TCF, BMP/TGFß and cytokine/chemokine pathway hits identified for CAP2 and ivermectin from the microarray data (D). Change is given in fold (positive or negative) and only reliable _at or *_*a_at probes are counted. For probe set ID see panel C in S7 Fig.

To identify WNT-TCF response blockers in a collection of natural compounds (Megabolite® MEGx), and scaffold-derived synthetic natural product analogues (Natdiverse^TM^, Natx), from Analyticon Discovery, we performed a layered screen for agents that mimic genetic blockade of TCF function with Luciferase reporters [6,7], WNT-TCF gene response signatures and BrdU analyses (S1 Text and S1 Fig). The sole confirmed hit (NP-002268) hereafter named CAP1, has a withanolide structure consisting of a steroidal ring core, an attached lactone ring and a sugar moiety (Fig 1A).

Searches in the Analyticon Discovery natural product database revealed the existence of two additional compounds (CAP2 and CAP3) with a similar scaffold (Fig 1A) as well as a more distantly related compound (CAP4; S2 Fig). CAP2 and CAP3, like CAP1, but not CAP4, were found to have repressive activity in luciferase assays and to repress WNT-TCF targets (S2 and S3 Figs).

Comparison of the effects of CAP1, CAP2 and CAP3 on CC14 primary human colon cancer cells [6,8,10,11] showed the higher potency of CAP2, which is the aglycone of CAP1 (Fig 1A): Analysis of fluorescence intensity of CC14-GFP^+^ cells, as a measure of overall cell mass and viability, treated with CAP1, CAP2, CAP3 or DMSO-only as control revealed that at 2.5μM all three CAP molecules had similar negative effects (panel C in S3 Fig). However, at 0.15μM only CAP2 was active (Fig 1B), and this was paralleled by the repression of the WNT-TCF targets *AXIN2*, *LGR5* and *cMYC* by CAP2, but not CAP1 (Fig 1B).

CAP3 belongs to the cardenolide family and exploration of FDA-approved cardenolides (e.g. oubain, digitoxin) and related compounds failed to reveal molecules that fully mimic the effects of CAP2 (S1 Text and S4 Fig).

### Concentration-dependent WNT-TCF target repression in multiple human cancer types and reversion by constitutively active TCF

CAP2 repressed WNT-TCF targets in multiple cancer types (Fig 1B and 1C) including all colon cancer cell lines tested. This included Ls174T cells harboring a gain of function *ßCATENIN* mutation, DLD1 and HT29 cells with loss of function mutations in *APC* (Fig 1C and not shown), as well as in three not cloned primary colon cancer cells: two from bowel tumors (CC14 and CC36) and one from a liver metastasis (mCC11) [6,8]. Whereas there were small context-dependent differences, the canonical and bona fide colon cancer WNT-TCF targets *AXIN2* and *LGR5* were generally repressed at 5μM CAP2 (batch 2). CAP2 treatment also repressed *AXIN2* and *LGR5* in human U87 and U251 glioblastoma cells, and *AXIN2* and *LEF1* in both MeWo human melanoma and H358 human lung cancer cells (since *LGR5* was not detected here), with WNT-TCF targets generally regulated in a dose-dependent manner (Fig 1C). This regulation was paralleled by increased Caspase 3^+^ apoptosis (panel A in S5 Fig).

To attempt to revert the effects of CAP2 we used a fusion between TCF and the potent transactivation domain of the viral VP16 protein, TCF^VP16^ [12]. Analyses in three colon cancer cell types revealed the epistatic rescue of the expression of *AXIN2* from CAP2 repression in cells with TCF^VP16^ [panel B in S5 Fig]. Rescue of the repression of the TCF targets *LGR5*, *cMYC* and *ASCL2* by CAP2 was also observed although there was variability between the cell types. TCF^VP16^ reversed the enhancement of *CDKN1A* by CAP2 (panel B in S5 Fig).

### Genome-wide transcriptional changes in human colon cancer cells

To test the effects of CAP2 on the transcriptome we performed microarray analyses with human colon cancer Ls174T cells as these have been previously used to determine changes driven by dnTCF activity [13]. Ls174T cells were treated with a separate batch of CAP2 (batch 3) at 5μM for 16h, and their transcriptome compared with that of control cells treated with DMSO under the same conditions. This dose was chosen for its consistent WNT-TCF target modulation in multiple cell types (Fig 1C, panel C in S5 Fig).

Global analyses in CAP2-treated Ls174T cells in triplicates using a 2-fold threshold over control with p<0.05 revealed cohorts of downregulated and upregulated probes (Fig 1D; S1 Text and S6 Fig), including the repression of a number of archetypal and direct TCF targets: *PTPRO* (19-fold), *LGR5* (15-fold), *cMYC* (10-fold) and *AXIN2* (4-fold) (Fig 1D blue dots, E).

### Comparison of CAP2 and ivermectin transcriptomes

We sought to compare transcriptomes of cells treated with CAP2 and ivermectin—a recently described WNT-TCF response blocker [6]—to define specificities and differential activities at similar doses. Genome-wide analysis of transcriptional changes in Ls174T cells treated with ivermectin revealed a smaller number of downregulated or upregulated probes in comparison to CAP2-treated cells, all over DMSO-treated controls (Fig 1D). Like for CAP2, probes for archetypal WNT-TCF targets (*PTPRO*, *LGR5*, *cMYC*, *AXIN2*) were strongly repressed (Fig 1D red dots; S1 Text and S6 Fig).

Analysis (S1 Text and S6 Fig) using Panther tools yielded similar overall distributions between CAP2 and ivermectin-treatments: they highlighted binding, nucleic acid binding and metabolic processes in both cases (S7 Fig). BMP/TGFß, WNT and chemokine/cytokine were identified as the top three affected pathways (Fig 1E, S7 Fig).

The effects of CAP2 and ivermectin might also be compounded by different secondary interactions. For instance, the expression of the genes encoding the WNT signaling promoting factor SRSF1 [14] and the WNT and BMP signaling repressive protein NOV [15] were downregulated and upregulated 21-fold, respectively, by CAP2. In contrast, *FILIP1L*, which has been suggested to repress WNT signaling [16], was upregulated 10-fold by ivermectin. Similarly, the upregulation of a number of interferon responsive genes by ivermectin (*ISG20*, *IFIT1*, *OASL*, *IRF9*, *IFI44*, *IFIT2;* S6 Fig*)* is intriguing since interferon treatment has been suggested to repress WNT-TCF signaling [17]. *DKK1* was consistently repressed by CAP2 treatment by 2-10-fold in Ls174T and DLD1 cells (not shown), indicating that the mechanism of action of CAP2 is not the derepression of this common antagonist.

### Global repression of TCF-regulated genes by CAP2 and avermectins

**Fig 2:**
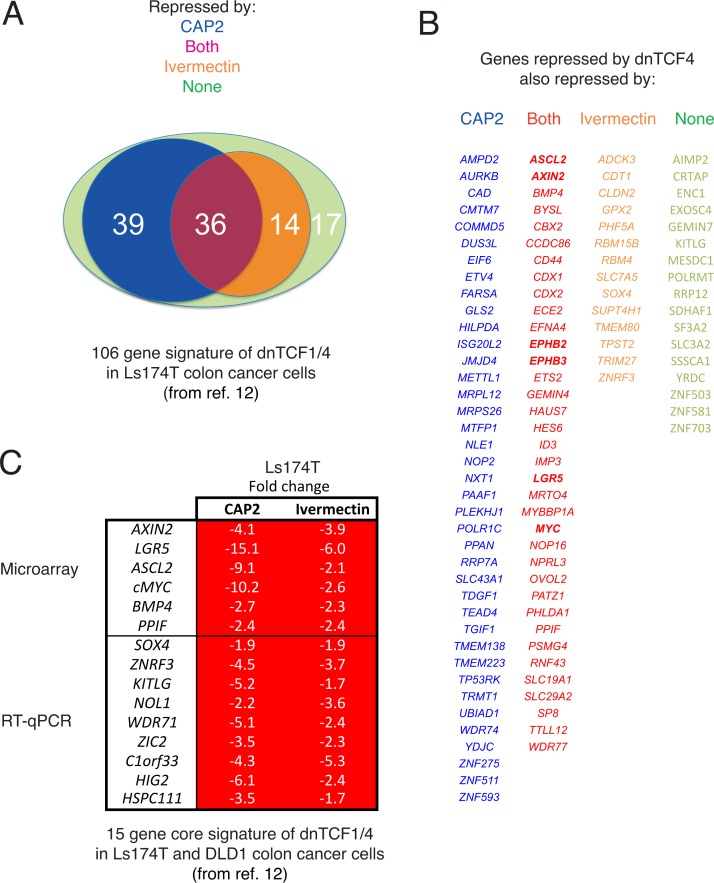
CAP2 and ivermectin regulate a large set of TCF-targets known to be repressed by dnTCF. A) Venn diagram representation of the overlap of 106 TCF-responsive genes in Ls174T cells [13] with the set repressed by CAP2 (75 genes), ivermectin (50 genes) or both (36 genes). 39 genes are repressed only by CAP2 and not by ivermectin, and 14 by ivermectin and not by CAP2. 17 genes are neither repressed by CAP2 nor ivermectin. B) Color-coded lists of genes in each class shown in (A). C) Heat map of the changes (in fold) observed after CAP2 or ivermectin treatment of Ls174T cells for a core set of 15 genes repressed by dnTCF1/4 in both DLD1 and Ls174T cells from [13]. The upper part is derived from the microarray data and the bottom was directly determined in cells treated as those used for the microarrays by RT-qPCR with specific primers. Whereas both CAP2 and ivermectin repress the entire set each shows gene-specific differences in the extent of the repression. All values are normalized over DMSO-treated controls.

To more directly analyze the effects of CAP2 and ivermectin on TCF targets, we used a published TCF target gene set in Ls174T cells [13] from which 106 genes could be detected in our microarray (above 75 units). CAP2 treatment repressed 70% of the gene set by 1.5-fold or more 16h post-treatment vs. 47% for ivermectin at the same dose (Fig 2A). Whereas this difference could be due in part to the kinetics of drug action, both drugs equally repressed a central set of 36 genes that included the canonical WNT-TCF targets *AXIN2*, *ASCL2*, *LGR5* and *cMYC* (Fig 2B).

We further analyzed the behavior of a core of 15 WNT-TCF targets that were found repressed by dnTCF in both Ls174T and DLD1 cells [13]. Since only a part of this previously described set was detected in the microarray data (using a cutoff of 75 detection units) we used RT-qPCR to complete the set. This 15-gene core TCF target set was fully repressed by both CAP2 and ivermectin under the same conditions (Fig 2C) although the degree of repression slightly varied.

A second avermectin, selamectin, showed similar profiles in the 106-gene signature, and displayed full repression of the 15-gene core signature (S1 Text and S8 Fig). The comparisons of the 106 gene set may yield underestimates of overlap since, for instance, the known WNT-TCF target gene *SOX4* was repressed in the core signature determined by rt-qPCR (Fig 2C), but not considered in the array results as it was below the used threshold (Fig 2A and 2B).

The present findings are striking since the chemical structures of macrocyclic lactones (ivermectin and selamectin) and steroidal lactones (CAP2) do not suggest simple interconversions or scaffold overlap. Such parallel effects raised the possibility that these different molecules could have similar activity requirements. Ivermectin is known to repress the protein levels of the TCF target CYCLIN D1 in an okadaic acid-sensitive manner, suggesting its action involves the protein phosphatases PP2A and/or PP1 [6]. Similarly, treatment of DLD1 cells with 5μM CAP2 (batch 3) resulted in a 2-fold decrease in the level of CYCLIN D1 and this repression was reversed by co-treatment with 15nM okadaic acid (panel A in S9 Fig).

### In vivo anti-tumor activity

The ability of CAP2 to inhibit tumor growth was tested in immunocompromised NUDE mice xenografted with the TCF-dependent colon cancer cell line DLD1. This cell line was chosen as it is dnTCF4-responsive in vivo: DLD1 xenograft growth is inhibited by both dnTCF4 and by systemic ivermectin treatments [6,10]. Analysis of gene expression changes in vitro validated DLD1 cells for the analysis in vivo since DLD1 cells displayed the same overall response to CAP2 treatment as Ls174T cells (panel B in S9 Fig).

**Fig 3:**
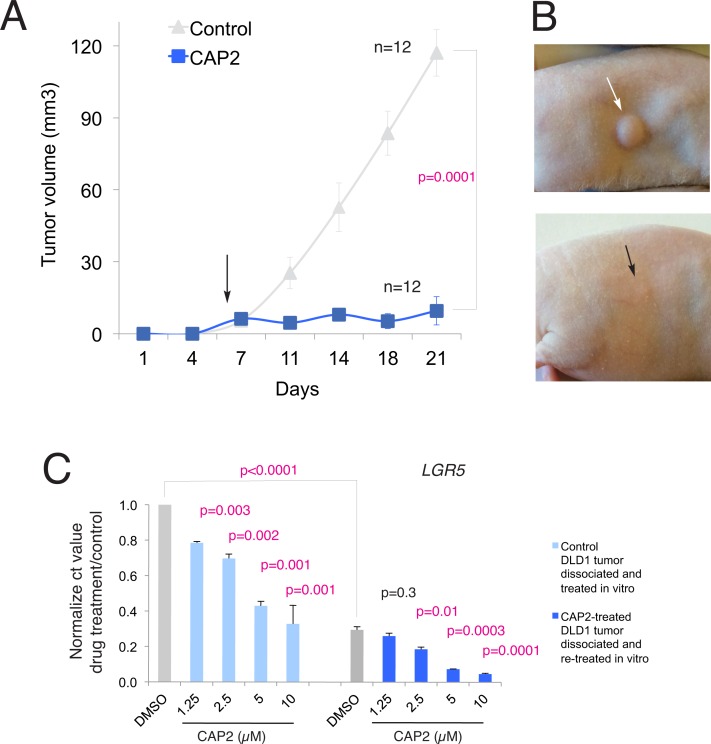
CAP2 inhibits DLD1 human colon cancer xenograft growth. A) Graph of tumor growth over time measuring tumor volume in control (cyclodextrin alone) DLD1 xenografts in Nude mice versus those receiving cyclodextrin-conjugated CAP2. The p value is given for the endpoint of the experiment noted by the bracket. n = number of tumors. B) Representative image of subcutaneous DLD1 xenograft tumor (arrows) growth seen in control mice (up) and those receiving CAP2 systemically administered via IP (bottom). C) Histograms of the repression of *LGR5* mRNA levels by CAP2 treatment in cells derived from control tumors (left) and those previously treated with CAP2 (right), both after dissociation and in vitro treatment for 16h with different concentrations of CAP2. Note the dose-dependent repression of *LGR5* levels in relation to the base of controls (gray bars), which are lower in DMSO-treated cells derived from CAP2 treated tumors in vivo than in control cells from control tumors. The level on DMSO-treated cells from control tumors is set to 1 and the other values (light blue for CAP2-treated cells from cyclodextrin only-treated control tumors or darker blue for CAP2-treated cells from CAP2-treated tumors) are set in reference to this. P values are in relation to the respective DMSO-treated controls (gray bars) unless noted by the bracket that shows the P value between in vivo pretreated with CAP2 and non-pretreated DMSO controls (gray bars).

DLD1-GFP^+^engrafted mice were treated with cyclodextrin-conjugated CAP2 at 10mg/kg via IP every second day starting at the time when tumors were palpable. Systemic delivery of CAP2, but not cyclodextrin carrier alone, resulted in the blockade of tumor growth without visible side effects in two independent experiments (Fig 3A and 3B).

Lack of general effects was supported by the finding that human 293T cells were not affected by CAP2 (batch3) treatment: the levels of *AXIN2*, *LGR5* and *CDKN1A* were statistically unchanged in triplicates after 16h treatment with 5μM CAP2 (*AXIN2*: 0.5μM: 1.1 fold over control, p = 0.8; 5μM: 0.7 fold, p = 0.1. *LGR5*: 0.5μM: 1.1 fold, p = 0.6; 5μM: 1.2 fold, p = 0.6. *CDKN1A*: 0.5μM: 0.6 fold, p = 0.3; 5μM: 1.4 fold, p = 1). Similarly, CAP2 did not affect BrdU incorporation in these cells (control: 45% BrdU^+^ cells of total DAPI^+^ cells; CAP2-treated cells: 46%).

### Long-lasting repression of WNT-TCF targets

Four DLD1 CAP2-treated regressed tumors were monitored after cessation of treatment to determine recurrence. After 90 days following drug removal only one tumor re-grew, suggesting a penetrant effect of CAP2.

Tumor regrowth, however, could be due in principle to insufficient drug dosage or to the development of drug resistance. The recurrent tumor was excised and the cells plated in vitro to test for the possible development of CAP2 resistance. One control in-vivo-cyclodextrin-treated tumor taken at 21d was similarly processed to serve as control. In vitro, treatment of tumor-derived cells with CAP2 resulted in a concentration-dependent repression of TCF targets below the levels of DMSO-treated control cells (S10 Fig), indicating the absence of drug resistance.

In vitro DMSO treatment of cells derived from in-vivo-CAP2-treated tumors resulted in TCF target levels that were lower than those observed in in-vitro DMSO-treated cells from in-vivo-cyclodextrin-treated tumors (S10 Fig), showing dose-dependent effects as exemplified by *LGR5* (Fig 3C). This consistent difference in the levels of several WNT-TCF targets raised the possibility that whereas tumor recurrence might be due to incomplete dosage, the recurring tumor harbored the memory of previous CAP2 treatments.

### Inhibition of cancer stem cell clonogenicity long after drug treatment

To directly test for the effects of long-lasting repression of WNT-TCF targets by CAP2 we focused on cancer stem cell clonogenicity, which is WNT-TCF-dependent [18]. DLD1 and Ls174T cells were first treated with CAP2 in 2D culture followed by removal of the drug, washing with PBS, and plating under non-adherent 3D clonogenic conditions for clonal spheroid growth in the absence of the drug. After primary growth, the spheroids were dissociated and replated as single cells for secondary and subsequent clonogenic tests, always in the absence of the drug. As negative control we used DMSO-treatment and as positive control we used treatment with ivermectin, which diminishes the clonogenicity of colon cancer stem cells [6].

**Fig 4:**
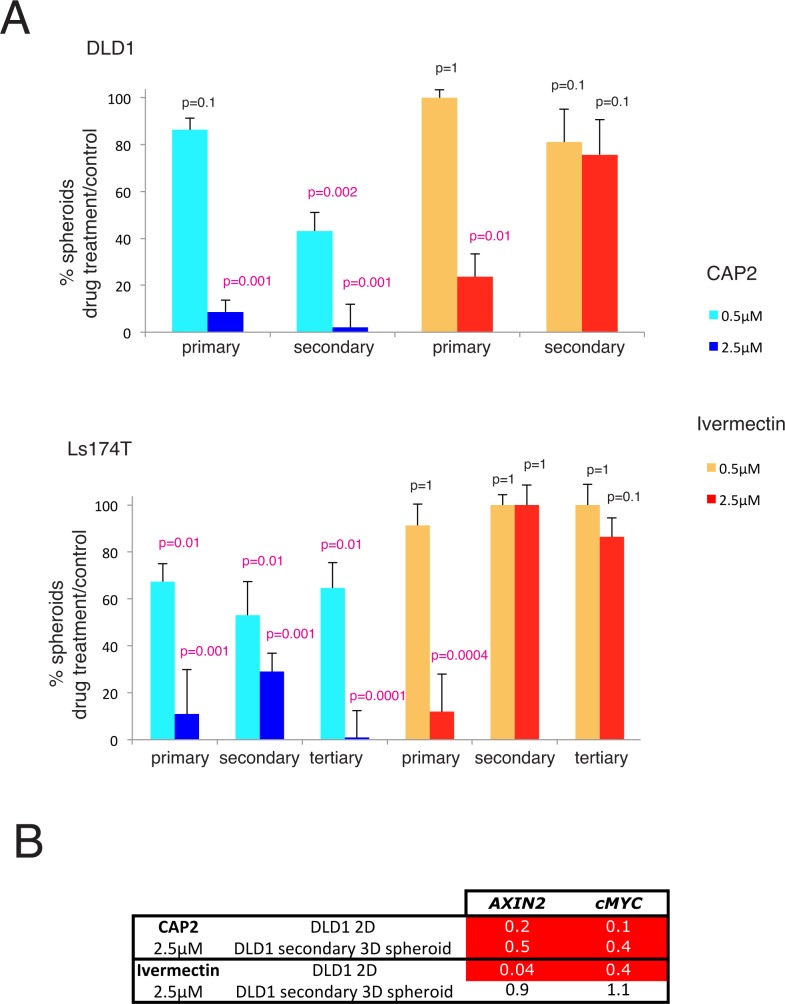
Long-lasting repression of stem cell clonogenic growth and of WNT-TCF targets by CAP2. A) Quantification of 3D clonal spheroids in primary, secondary or tertiary assays from DLD1 (upper panel) or Ls174T (bottom panel) cells previously treated in 2D culture with CAP2 (blue) or ivermectin (red) at 0.5μM (light blue and orange) and 2.5μM (dark blue or red), as compared with control (100%, not shown). Spheroids were not treated with drugs. CAP2 treatment in 2D results in a decrease in primary 3D clonogenic spheroids in both cell types, arguing for a decrease in clonogenic stem cells. Similar results were obtained with ivermectin [6]. However, secondary and tertiary clonal spheroid assays reveal that the repression imposed by CAP2 is long-lasting, in contrast with that imposed by treatment with ivermectin. CAP2 pretreatment of Ls174T cells at 5μM leads to the exhaustion of clonogenic cancer stem cells after three passes. In the case of ivermectin, clonal frequencies recover and become identical to those obtained from DMSO-pretreated control cells, the value of which is here equated to 100 (not shown). p values are in relation to DMSO controls. B) Heat map of showing the repression of normalized *AXIN2* and *cMYC* levels, used here as direct targets of the WNT-TCF pathway, in DLD1 cells treated in vitro and in secondary spheroids derived from these 2D treated cells. Note that CAP2 repression is long-lasting as it is evident in secondary spheroids. In contrast, the repression imposed by treatment with ivermectin is evident in 2D but absent in secondary spheroids.

Quantification revealed a dose-dependent decrease in primary DLD1 or Ls174T spheroids after treatment with either CAP2 or ivermectin in 2D, normalized over DMSO controls (Fig 4A). The frequency of secondary clonogenic spheroids made from primary spheroids in the absence of drugs was like that of DMSO-treated controls for the ivermectin pretreated cells, as expected since the drug was no longer present [6]. Surprisingly, this was not the case for those pretreated with CAP2. The percentage of spheroids derived from CAP2 treated cells continued to decrease in the long-term absence of drug as secondary and tertiary clonogenic spheroids also showed diminished frequencies and was close to zero for tertiary LS174T 3D spheroids derived from cells pretreated in 2D with 2.5μM CAP2 (Fig 4A).

Treatment in 2D culture resulted in the concentration-dependent repression of WNT-TCF targets by CAP2 and ivermectin after 16h (Fig 4B), as expected. However, analyses of their expression in secondary spheroids revealed sustained repression in CAP2—but not in ivermectin-pretreated samples—as compared with DMSO controls (Fig 4B).

### Modulation of the expression levels of a large cohort of chromatin components and remodelers

**Fig 5:**
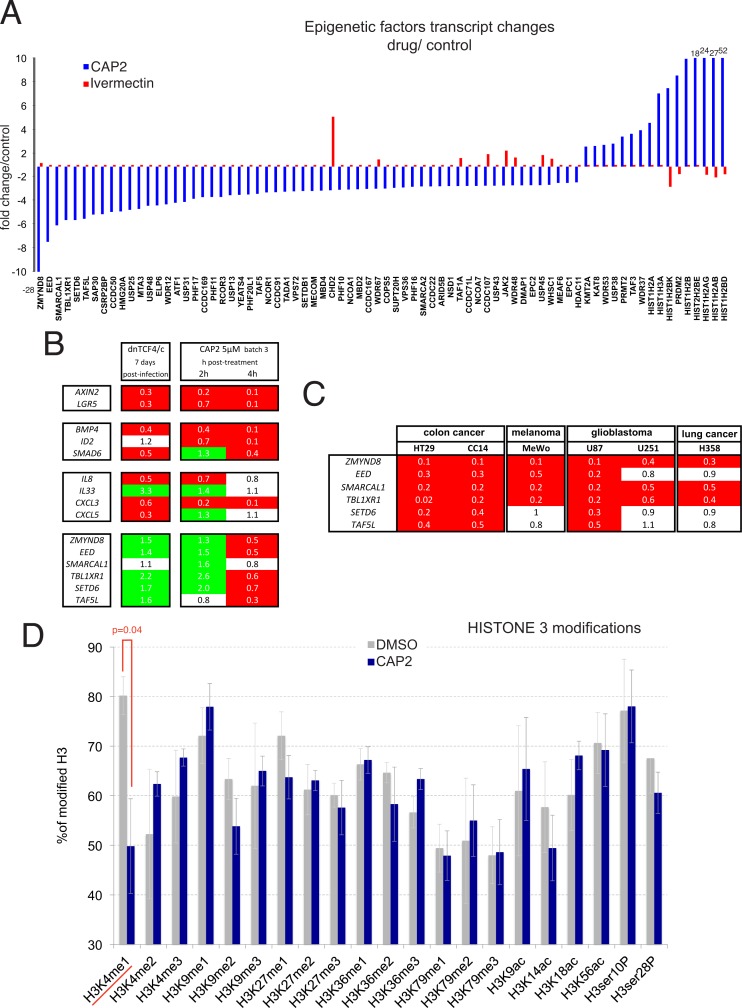
Regulation of chromatin components and remodelers by CAP2. A) Genomic analysis of transcriptomic changes in Ls174T cells imposed by CAP2 but not by ivermectin on an epigenetic gene set revealed large repression or increases in the levels of chromatin remodelers and components. The x-axis denotes the genes (using only reliable _a or _a*_*at probes) with the associated fold change in relation with DMSO-treated controls, both for CAP2 (blue) and for ivermectin (red) treatments in the y-axis. Changes off scale are written near the respective column. B) Modulation of selected WNT-TCF, BMP, cytokine/chemokine and epigenetic regulator genes by stable dnTCF expression (left) and after 2 or 4h following CAP2 treatment as indicated (right). C) Heat maps of the changes in gene expression after CAP2 treatment (0.5μM batch 4 for 16h) in different human cancer cells as indicated. All values in the heat maps in (B,C) show normalized ratios over DMSO controls. D) Histogram showing CAP2-driven changes in HISTONE 3 modifications. The average of four assays is shown for control DMSO-only treated Ls174T cells and CAP2-treated sibling cells as described in Materials and Methods. The only significative change (p<0.05) was detected in the levels of monomethylated lysine 4.

The memory imposed by CAP2 on clonogenicity and WNT-TCF responses could suggest epigenetic changes. To begin to test this possibility, we mined the transcriptome for CAP2-induced expression alterations (over DMSO controls) not mimicked by ivermectin treatment, using the public Reactome database for a chromatin organization cluster that we expanded to include related family members (94 probes). This analysis (using only specific _a and _a_at probes) revealed a cohort of 75 genes (about 80%) with CAP2-specific expression changes by 3-fold or more (Fig 5A).

Of these, *ZMYND8*, *EED*, *SMARCAL1*, *TBL1XR1*, *SETD6*, *TAF5L*, *SAP30*, *CSRP2BP*, *CCDC50*, *HMG20A*, *USP25*, and *MTA3* were downregulated 5-fold or more. Conversely several *HISTONE* genes, *PRDM2 (RIZ)*, *WDR37*, *TAF3* and *PRMT2* were upregulated 5-fold or more (Fig 5A).

### Specificity within the withanolide family

The unusual ability of CAP2, which represents withanolide F, to repress WNT-TCF responses in human cancer cells together with its ability to alter epigenetic aspects of these cells drove us to test two related molecules for their mimicry of its effects. Here we have tested the modulation of selected WNT-TCF, cytokine and BMP signature markers, as well as the expression of the three most repressed genes by CAP2 encoding epigenetic regulators in human Ls174T colon cancer cells by withanolide A and withanolide B. None reproduced the signature changes of CAP2 (S11 Fig).

### Potency and primacy of WNT-TCF repression and evidence for a WNT-TCF-independent action of CAP2 on epigenetic regulators

The multiple pathways regulated by CAP2 raised the question of the primacy of WNT-TCF repression. To further compare CAP2 action with that of dnTCF, we analyzed 9 components of the top three repressed Panther pathways, WNT-TCF, BMP/TGFß and chemokine/cytokine, and the top 6 repressed epigenetic regulators. Analyses of the steady-state effects of direct TCF repression by dnTCF in DLD1 cells, used for in vivo experiments, confirmed the repression of *AXIN2* and *LGR5* (Fig 5B). DnTCF also repressed the levels of *BMP4*, *SMAD6*, *IL8*, *CXCL3 and CXCL5* (Fig 5B). DnTCF did not repress *ZMYND8*, *EED*, *SMARCAL1*, *TBL1XR1*, *TAF5L* or *SETD6* and instead it enhanced the expression of all but that of *SMARCAL1* (Fig 5B).

A time course of CAP2 treatment for 2 and 4 hours (at 5μM batch 3 in Ls174T cells used for the transcriptomic analyses) revealed that *AXIN2* and *LGR5* were already repressed by these early times (Fig 5B). *BMP4* and *ID2* were also repressed by 2h (Fig 5B). Similarly, *CXCL3* and *IL8* were already repressed at 2h (Fig 5B). At 4h only *CXCL3* was repressed (Fig 5B). None of the 6 epigenetic regulators tested were repressed at 2h: instead all but *TAF5L* were transiently enhanced at 2h, largely mimicking the effects of dnTCF, and all but *SMARCAL1* were repressed at 4h (Fig 5B).

Resupply of CAP2 (batch 4) revealed that it was more active than CAP2 (batch 3) kept in the lab for a few months at -20°C (batch 3), arguing that it might be partly labile: The transcriptional effects on selected targets at 0.5μM CAP2 batch 4 were similar to those obtained with 5μM of CAP2 batch 3 (not shown). Re-analysis of target regulation at different concentrations revealed activity of CAP2 batch 4 at 1nM (panel A in S12 Fig): After 16h treatment we could detect the repression of *LGR5* at 1nM, and of *LGR5* and *AXIN2* at 100nM CAP2. As comparison, DMSO-treated cells or cells treated with a fresh batch of CAP1 at the same doses did not modify the levels of *LGR5* or *AXIN*2 (not shown). 1nM CAP2 also generally repressed the other highlighted pathways, including BMP, chemokine/cytokine and epigenetic regulators, and all were repressed at 500nM (S12 Fig).

Like the repression of WNT-TCF responses, the repression of epigenetic regulators may have general components as it was also detected with cell type specific variations after 500nM treatment with CAP2 (batch 4) for 16h in CC14 and HT29 human colon cancer, MeWo human melanoma, U87 and U251 human glioma, and H358 lung cancer cells (Fig 5C).

### CAP2 represses the global levels of H3K4Me1

Following the changes observed in the levels of expression of epigenetic components and modulators by CAP2 treatment, we asked if CAP2 could change specific HISTONE marks by analyzing multiple HISTONE H3 modifications in DLD1 cells treated with CAP2 (5μM of batch 3) vs. control DMSO-treated cells. Most modifications showed little variation after normalization to total HISTONE H3 levels following treatment with DMSO or ivermectin (Fig 5D and not shown). However, mono-methylated lysine 4 (H3K4Me1) was significantly (p<0.05) reduced by 40% on average by CAP2 treatment (Fig 5D).

## Discussion

Our validated hit, CAP1, is a withanolide steroidal lactone glycoside previously identified as coagulin-L from *Withania coagulans* [19, 20]. It has been suggested to act as an adipogenic differentiation blocker perhaps enhancing WNT levels [20], as well as to inhibit both NOS production and NFkB signaling in the micromolar range [19]. Our lead, CAP2, is the aglycone of CAP1 and represents withanolide F, which was initially identified from the leaves of *Withania adpressa* and reported to have cytotoxic activity [9,21].

Assigning precise functions to different withanolides, however, is difficult. This is due in part to the finding that individual compounds can have multiple targets. For example, some have been reported to bind ANNEXIN II, ßTUBULIN, HSP90, and IKKß [22–24]. Defining the main function of a natural drug can also be confounded by the fact that single plant small molecules can have multiple effects on animal cells. For instance, extracts of producing plants (e.g. from *Withania somnifera*) have been used in Ayurveda for multiple indications [25] and withanolides have been reported to be cytotoxic, promote apoptosis and affect multiple cellular pathways including the blockade of AKT (e.g., [26–30]).

In this case, inhibition of AKT function can lead to GSK3ß activation and to WNT-TCF pathway blockade [26]. However, withanolide F seems to function differently, possibly acting downstream of GSK3ß since the N’ΔßCATENIN form we have used in our TCF reporter assays is predicted to be insensitive to GSK3ß modulation.

Comparison with a previously reported WNT-TCF response blocker, ivermectin, reveals a striking parallel in their global repression of WNT-TCF response genes. A 15-gene TCF core signature is fully repressed by both drugs, and they repress a large percentage of an Ls174T dnTCF-repressed 106-gene cohort [13]. Both withanolide F and ivermectin thus appear to be potent WNT-TCF response blockers in human cancer cells.

It may seem surprising that these different plant and bacteria WNT-TCF response blockers also repress other pathways, including chemokine/cytokine and BMP/TGFß. However, we show that these two are at least partially subordinate to WNT-TCF signaling. Notably, their repression may further help the effects of direct WNT-TCF blockade through common secondary loops. For instance, downregulation of BMP signaling in human colon cancer cells in vivo is expected to lead to tumor inhibition [31], and repression of *ID* genes is expected to contribute to the depletion of cancer stem cells [32]. Repression of multiple cytokine/chemokine encoding genes might be related to the large induction of *ATF3* by both drugs and might possibly parallel non-cell-autonomous events of chemokines in melanoma [33]. Other secondary effects further enhancing WNT-TCF repression that diverge between withanolide F and ivermectin may involve upregulated *NOV* and downregulated *SRSF1* by withanolide F and upregulated interferon responses by ivermectin. The existence of multiple modes of action [34] may thus enrich the effectiveness and desirability of withanolide F.

Since repression of endogenous canonical WNT-TCF signaling in the intestine, which is essential for proper crypt regeneration, is expected to cause weight loss and eventual death [35], it is striking that neither treatment with withanolide F nor with ivermectin [6] cause overt side effects in mice. Withanolide F, like ivermectin, could thus preferentially affect in vivo pathologically active over homeostatically normal canonical WNT signaling. How this may take place is unclear although we have previously suggested a possible mechanism for ivermectin involving protein phosphatases [6], which may also be operative in the case of withanolide F. Indeed, the actions of ivermectin and withanolide F are antagonized by okadaic acid treatment and appear to alter phosphatase levels as they upregulate the PP1 subunit *PPP1R10* by 3- and 2-fold and repress the PP2A regulatory subunit *PPP2R1B* by 6- and 2-fold, respectively. Alternatively, these drugs could affect other context-specific co-factors that promote WNT-TCF function in cancer cells since without such input, nuclear ßCATENIN binding to TCF target sites per se is insufficient to promote target gene expression [36].

Critically, withanolide F, but not ivermectin, induces long-lasting WNT-TCF pathway silencing in clonogenic cancer stem cells and long-lasting decreased self-renewal. Moreover, withanolide F modulates the expression of several genes involved in chromatin function and remodeling. While it remains to be determined which of these genes are critical, they include the bromodomain factor ZMYND8 involved in DNA repair [37] and implicated in cancer as a fusion protein [38]; the key Polycomb repressive complex 2 (PRC2) component EED, which associates with EZH2 (which trimethylates H3K27 causing gene silencing) and also functions in the coordination of PRC complexes [39]; the SWI/SNF chromatin remodeling factor SMARCAL1 involved in genome integrity (e.g., [40]); and multiple HISTONE genes.

Global analysis of HISTONE 3 marks reveal that CAP2, but not ivermectin, treatment of human colon cancer cells decreases overall H3K4me1 levels, supporting an effect of CAP2 on the epigenetic landscape, the reduction of a mark associated with active enhancers and the repression of associated genes [41]. Moreover, since H3K4Me1 has been also associated to hypomethylated DNA regions during aging [42], it is tempting to speculate that withanolide F treatment might lead to gene repression by hypermethylation and loss of enhancer activity. Future analyses should untangle how the repression of WNT-TCF signaling responses by CAP2, the modulation of epigenetic regulators and changes in individual epigenetic marks are connected in specific transcriptional units (e.g., [43–45]).

The long-lasting epigenetic memory that withanolide F imposes on human cancer cells represents a novel paradigm and offers a unique opportunity for prolonged WNT-TCF pathway silencing following restricted treatment, leading to cancer stem cell exhaustion and tumor regression. Such strategy should minimize possible side effects. More generally, this work reveals the possibility that signaling responses may be permanently shut down in cancer cells via epigenetic alterations driven by limited treatments with small molecules.

## Supporting Information

S1 Text Supplementary results and discussion. (DOCX)

S1 Table PCR primers used. (DOCX)

S1 Fig Primary screen for WNT-TCF antagonists and identification of hits. (EPS)

S2 Fig Structures and activities of CAP analogues. (EPS)

S3 Fig Effects of CAP molecules on WNT-TCF reporters, epithelial CC14 colon cancer cell morphology and effects of CAP1, 2 and 3 at 2.5μM. (EPS)

S4 Fig Effects of cardenolides on WNT-TCF response genes in Ls174T cells. (EPS)

S5 Fig Apoptotic, rescue and general effects of CAP2. (EPS)

S6 Fig Top microarray changes in gene expression. (PDF)

S7 Fig Analysis of transcriptomic changes induced by CAP2 and Ivermectin on human Ls174T colon cancer cells with Panther tools. (EPS)

S8 Fig Overlapping effects of selamectin, ivermectin and CAP2 as WNT-TCF response blockers. (EPS)

S9 Fig Okadaic acid sensitivity and gene regulation by CAP2. (EPS)

S10 Fig WNT-TCF gene expression changes after CAP2 treatment in vivo and in vitro. (EPS)

S11 Fig Withanolides A and B do not reproduce the effects of CAP2. (EPS)

S12 Fig Concentration and time effects of CAP2 treatments and regulation of *GLI1*. (EPS)
