# Supplementary material for: Long-Lasting WNT-TCF Response Blocking and Epigenetic Modifying Activities of Withanolide F in Human Cancer Cells
Source: PLoS One. 2016 Dec 14;11(12):e0168170. doi: 10.1371/journal.pone.0168170 (PMC5156407; doi:10.1371/journal.pone.0168170)
Supplement: S1 Text — (DOCX) [file pone.0168170.s001.docx]

**Supplementary data**

**Supplementary text:**

**Small molecule screen for WNT-TCF response blockers**

We used a TCF-binding site driving Firefly luciferase reporter in human 293T cells [1], utilizing dominant-negative (dn)TCF4 at every step as a genetic benchmark [2]. In this experimental setting, the expression of N-terminally truncated, APC-insensitive βCATENIN (N’∆βCATENIN), and of N’∆βCATENIN plus dnTCF4, gave a 5-fold dynamic range (S1A Fig.). All steps of the screen included normalization with TCF-independent Renilla luciferase controls, and dual luciferase activities were given as ratios of Firefly over Renilla values (S1A,B Fig.). Molecules scoring below the levels afforded by dnTCF were considered non-specific or toxic (S1B Fig.).

Testing 3076 natural products and 2468 scaffold library molecules in duplicate at 10µM yielded 15 compounds from the first and 14 from the latter that reduced Renilla-normalized levels of TCF-driven Firefly luciferase by 40% or more after 16h (S1B,C Fig. and not shown). These were then tested for the transcriptional modulation of a restricted TCF signature in human colon cancer Ls174T cells (S1C Fig.) that included the direct targets *AXIN2* and *cMYC*, which should be repressed by WNT-TCF response antagonists, and *CDKN1A* (*p21*), which should be induced. Only two natural compounds repressing the luciferase reporter by >40% also repressed *AXIN2* and *cMYC* mRNA levels by >30%, and concomitantly boosted *CDKN1A* levels by 2-fold or more (Fig. S1C Fig.). We highlighted these two compounds (1092 and 454) plus two additional natural compounds that either boosted *CDKN1A* levels significantly (1083) or repressed *cMYC* (1273).

Eleven natural compounds including the four mentioned above were then evaluated for an inhibitory effect on Ls174T cell proliferation, as measured by BrdU incorporation. Of these, seven reduced BrdU incorporation by > 50% at 10µM after 16h (purple boxes in S1C Fig.). BrdU incorporation supported the choice of the top 4 compounds (1092, 454,1273 and 1083; highlighted in blue in S1C Fig.) based on luciferase reporter data and gene expression analyses. These 4 compounds represent in a discovery rate of 0.07% from the chemical libraries used in the primary and secondary screens.

**Steroidal lactones and related molecules as WNT-TCF response blockers in human colon cancer cells**

The four natural product hits represented different chemical spaces (S1D, 1A Figs.) and all four were further tested for the modulation of an extended WNT-TCF regulated gene signature including *LGR5, EFNB1, EPHB2* and *EPHB3* and compared them to the modulation of target gene expression by dnTCF4 (S1E Fig.). Compounds 1092, 454 and 1273 showed a repeated coherent modulation of targets, mimicking the effects of pathway blockade by dnTCF4, with 1092 being the top hit, followed by 454 and 1273 (S1E Fig.). Compound 1083 showed only partial transcriptional modulation and was abandoned.

The three remaining hits were then retested with an independent second batch of each compound purified to >98%. Only one of these second batches showed activity in repressing *AXIN2*, *LGR5* and *cMYC* (not shown). This compound, 454 (Analyticon Discovery molecule NP-002268, hereafter named CAP1), thus represents the sole validated hit of the screen. Efforts to isolate minor active compounds in preparations of 1092 were unsuccessful (not shown).

Searching analogs of CAP1 in the Analyticon Discovery libraries yielded NP-002271 (hereafter CAP2), which is the aglycon of CAP1 (Fig. 1A, S2A Fig.); NP-010989 (hereafter CAP3), which is a cardenolide cardiac glycoside with a different sugar moiety and a different type of (γ) lactone ring (Fig. 1A, S2A Fig.); and NP-007960 (hereafter CAP4), which has a sugar moiety at the same position as CAP1 and CAP3 but an alkyl chain and two sugars at different ends (S2A Fig.).

Dual luciferase reporter assays in 293T revealed that CAP1, CAP2 and CAP3 had similar repressive activities whereas CAP4 was inactive as compared with DMSO-only controls (S3A Fig.). Similarly, gene expression analyses of the effects of 10µM drug treatments for 16h on Ls174T colon cancer cells revealed the WNT-TCF response blocking activities of CAP1, CAP2 and CAP3, whereas CAP4 was largely ineffective (S2B Fig.).

The typical epithelial island morphology of CC14 cells was maintained after 24h treatment with 1µM CAP1 or CAP3 but this was totally disrupted after treatment with the same dose of CAP2 (S3B Fig.). Similar phenotypes were only observed at 10µM of CAP1 or 5µM of CAP3 (S3B Fig.).

The inactivity of CAP4 indicated that not all steroidal lactones or related molecules with similar scaffolds share the WNT-TCF response inhibitory activity of CAP1, CAP2 and CAP3.

**Clinically approved and related cardenolides do not mimic the WNT-TCF blocking activity of CAP molecules**

Given the regulation of WNT-TCF targets by CAP3 and its cardenolide structure, we aimed to explore this family for clinically approved compounds that may harbor WNT-TCF response blocking activities, including ouabain, as well as digoxin and digitoxin from *Digitalis.* These molecules are used for the prevention of congestive heart failure, although a number of cardenolides have also been shown to have anti-cancer effects and to engage an immunogenic death [e.g. 3-7],

Measurements of gene expression by rt-qPCR in Ls174T colon cancer cells treated with 0.5µM of digitoxin, digoxin, gitoxigenin, ouabain, neriifolin, cymarin, convallatoxin or oleandrin for 16h resulted in changes in WNT-TCF target expression over DMSO-treated controls (S4 Fig.): Only digoxin and digitoxin repressed *AXIN2*, whereas only gitoxigenin and oleandrin repressed *LGR5*. Cymarin was ineffective and the five other drugs enhanced *LGR5* expression; *cMYC* was not repressed by digoxin or cymarin and *CDKN1A* was enhanced by digoxin, digitoxin, ouabain and neriifolin but repressed by gitoxigenin and convallatoxin. Digoxin and digitoxin might harbor some aspects of a desired antagonist since they repressed *AXIN2* and enhanced *CDKN1A*. Conversely, convallatoxin might have partial agonist activity as it enhanced *AXIN2* and *LGR5* and repressed *CDKN1A*, although it also repressed *cMYC* (S4 Fig.). However, none of these drugs reproduced the coordinated modulation of WNT-TCF targets we observed with CAP2 or dnTCF.

**CAP2 enhances apoptosis**

Analyses of activated cleaved Caspase3 labeling as a marker of apoptosis revealed an increase number of labeled cells by FACS starting at 2.5µM of CAP2 for 16h as compared with DMSO-treated Ls174T and DLD1 cells (S5A Fig.). The level at which we first detected a significant increase in apoptosis (at 2.5µM CAP2) was higher than that which changes cell proliferation assessed by BrdU incorporation (1.25µM CAP2) and gene expression changes (see below), suggesting that the effects we observed with CAP2 at low doses were unlikely due to simple apoptosis.

**Genome-wide transcript changes in human colon cancer cells by CAP2**

Overall, 532 genes were upregulated by 5-fold or more, of which 9 were up by 20-fold or more (*HIST1H2BD, ALDH8A1, HIST1H2AB, TTC18, HIST1H2AG, HIST1H2AM, NCALD, ERVK3-2, NOV*). 110 genes were downregulated by 5-fold or more, of which 11 were down by 20-fold or more (*SYNCRIP, NMI, ZMYND8, SLC16A14, VAV3, RFXAP, KIT, SRSF1, SPC25, PPIL1, ARMCX5*) (S6 Fig.).

Other notable repressed genes included *MDM2*, encoding a p53-interacting and inhibiting protein was inhibited 13-fold; *NMI* (down 32-fold) encoding a MYC and STAT interacting protein; *KIT* (down 23-fold) and *BCL2* (down 5-fold) involved in cell survival; and *YAP* (down 6-fold) which has been suggested to be WNT-TCF regulated [8].

We also found the 5-fold repression of *IFT74, IFT81, KIF21A* and *KIF11*, which are involved in cell migration, intracellular transport and/or ciliogenesis [e.g. 9-11]. This raised the possibility that the activity of cilia-dependent pathways, such as HEDGEHOG (HH)-GLI [e.g. 12], may be compromised. Whereas *GLI1* levels were below the level of reliable detection in the arrays, measurement by rt-qPCR revealed a complex regulation of *GLI1* by CAP2, with its levels being sensitive to dose and time of treatment (S12B Fig.).

**Comparison of CAP2 and ivermectin transcriptomes**

Overall, ivermectin treatment resulted in 48 genes repressed by 5-fold or more (S6 Fig.), of which ten were repressed by 10-fold or more (*MT4, CXCL5, FGFBP1, UGT2B15, HMGB3, PTGDR, PTPRO, OLFM4, SMAD6, C10Orf99*) including 3 known WNT-TCF targets (*PTGDR, PTPRO, OLFM4,* down 13 to 15-fold). Conversely, ivermectin upregulated 71 genes by more than 5-fold (S6 Fig.), of which 15 were up by more than 10-fold (*CASP5, RAB39B, ATF3, DDIT3, BEX2, ISG20, IFIT1, OASL, IRF9, PEAR1, IL1A, CDKN2B, EGR1, FILIP1L, GADD45A*) highlighting DNA damage and cell cycle regulation (*ATF3, DDIT3, CASP5, CDKN2B, EGR1, GADD45A*) as well as interferon related responses (*ISG20, IFIT1, OASL, IRF9*). Induction of interferon-responsive genes also raises the possibility that the drugs may activate immune or anti-viral like responses [e.g. 13,14] although WNT-TCF may also directly induce aspects of interferon signaling [15].

Comparison of the top downregulated probes by 5-fold or more in CAP2- (532 genes represented by 732 probes) versus in ivermectin-treated (48 genes, 62 probes) cells revealed 14 genes repressed in both cases (*MYB, PTPRO, LGR5, PTDGR, C2CD4A, PLA2G3, FGFBP1, OR51E1, ID2, ID4, SMAD6, MT4, MCM3 and HNRNPU*) highlighting the WNT (*PTPRO, LGR5, PTDGR*) and BMP pathways (*ID2, ID4, SMAD6*) (S6, S7 Figs.). We also note that MYB has been shown to act positively on WNT-TCF targets in breast cancer [16]. Conversely, analysis of the upregulated probes by 5-fold or more in both CAP2- (110 genes, 149 probes) and ivermectin-treated cells (71 genes, 107 probes) failed to reveal any particular Panther pathway and only 3 genes were upregulated at this level by both treatments: *ATF3, EGR1* and *MIR22*, the latter implicated in histone modifications in cancer [17].

Each drug also had specific effects since 32 genes were differentially regulated (up and down or down and up with the two drugs) at 3-fold or more, but only one, *C8ORF4,* at more than 5-fold (9-fold up by ivermectin and 8-fold down by CAP2). The product of this gene, TC1, appears to have context-dependent functions as it has been implicated as a positive regulator of WNT-TCF signaling [18], but also as a repressor of liver cancer stem cell self-renewal through inhibition of NOTCH signaling [19].

**Comparison with selamectin**

The data also raised the possibility that analysis of the WNT-TCF blocking activity of the related avermectin family compound selamectin could help to further differentiate activities of these blockers. Selamectin was previously found to harbor WNT-TCF response blocking function acting at the nanomolar range [2]. In the present study, however, three different batches of Selamectin were tested and all were found to harbor the expected WNT-TCF blocking activity but at the micromolar range (not shown). Analyses of transcriptomic changes afforded in Ls174T cells by 5µM selamectin treatment for 16h (the same dose used for CAP2 or ivermectin using the same microarray), showed a clear overlap (S8 Fig.) with the 106-gene set reported for dnTCF [20]. A large core of canonical targets were repressed by CAP2, ivermectin and selamectin whereas there was a single gene repressed by dnTCF in LS174T cells that was solely repressed by selamectin at the dose used (S8 Fig.). Surprisingly, there was a similar overlap of inhibited dnTCF sensitive genes between the three compounds. The partial overlap could be due, in part, to dose effects: for example, *SOX4* was repressed by CAP2 by 1.9-fold but was absent from the list used with a cutoff of 2-fold for the Venn diagram overlap comparisons (S8 Fig.). It is thus possible all three compounds reliable repress WNT-TCF targets. Indeed, as with ivermectin and CAP2, selamectin fully repressed the 15-gene dnTCF core signature common to both Ls174T and DLD1 cells (S8C Fig.) [20]. Together, these results argue that CAP2 parallels the WNT-TCF repressing activities of avermectin macrocyclic lactones, and that these macrocyclic and steroidal lactones have largely overlapping but not identical effects.

**Supplementary references**

1          Barker N, Clevers H. Mining the Wnt pathway for cancer therapeutics. Nat Rev Drug Discov. 2006;5(12):997-1014.

2          Melotti A, Mas C, Kuciak M, Lorente-Trigos A, Borges I, Ruiz i Altaba A. The river blindness drug Ivermectin and related macrocyclic lactones inhibit WNT-TCF pathway responses in human cancer. EMBO Mol Med. 2014;6(10):1263-78.

3          Prassas I, Diamandis EP. Novel therapeutic applications of cardiac glycosides. Nat Rev Drug Discov. 2008;7(11):926-35.

4          Prassas I, Karagiannis GS, Batruch I, Dimitromanolakis A, Datti A, Diamandis EP. Digitoxin-induced cytotoxicity in cancer cells is mediated through distinct kinase and interferon signaling networks. Mol Cancer Ther. 2011;10(11):2083-93.

5          Simpson CD, Mawji IA, Anyiwe K, Williams MA, Wang X, Venugopal AL, et al. Inhibition of the sodium potassium adenosine triphosphatase pump sensitizes cancer cells to anoikis and prevents distant tumor formation. Cancer Res. 2009;69(7):2739-47.

6          Antczak C, Kloepping C, Radu C, Genski T, Müller-Kuhrt L, Siems K, et al. Revisiting old drugs as novel agents for retinoblastoma: in vitro and in vivo antitumor activity of cardenolides.  Invest Ophthalmol Vis Sci. 2009; 50(7):3065-73.

7          Menger L, Vacchelli E, Adjemian S, Martins I, Ma Y, Shen S,et al. Cardiac glycosides exert anticancer effects by inducing immunogenic cell death. Science Transl. Med. 2012;4(143),143ra99.

8          Konsavage WM Jr, Kyler SL, Rennoll SA, Jin G, Yochum GS. Wnt/b-catenin signaling regulates Yes-associated protein (YAP) gene expression in colorectal carcinoma cells. J Biol Chem. 2012;287(15): 11730-9.

9          Bhogaraju S, Cajanek L, Fort C, Blisnick T, Weber K, Taschner M, et al. Molecular basis of tubulin transport within the cilium by IFT74 and IFT81. Science. 2013;341(6149):1009-12.
10       Niwa S. Kinesin superfamily proteins and the regulation of microtubule dynamics in morphogenesis. Anat Sci Int. 2015;90(1):1-6.

11       Venere M, Horbinski C, Crish JF, Jin X, Vasanji A, Major J, et al. The mitotic kinesin KIF11 is a driver of invasion, proliferation, and self-renewal in glioblastoma. Sci Transl Med. 2015;7(304):304ra143.

12       Goetz SC, Anderson KV. The primary cilium: a signalling centre during vertebrate development. Nat Rev Genet. 2010;11(5):331-44.

13       Hayward SD, Liu J, Fujimuro M. Notch and Wnt signaling: mimicry and manipulation by gamma herpesviruses.  Sci STKE. 2006;(335):re4.

14       Shapira SD, Gat-Viks I, Shum BO, Dricot A, de Grace MM, Wu L, et al. A physical and regulatory map of host-influenza interactions reveals pathways in H1N1 infection. Cell. 2009;139(7):1255-67.

 15       Marcato V, Luron L, Laqueuvre LM, Simon D, Mansuroglu Z, Flamand M, et al. β-Catenin Upregulates the Constitutive and Virus-Induced Transcriptional Capacity of the Interferon Beta Promoter through T-Cell Factor Binding Sites. Mol Cell Biol. 2015;36(1):13-29.

 16       Li Y, Jin K, van Pelt GW, van Dam H, Yu X, Mesker WE, et al. c-Myb Enhances Breast Cancer Invasion and Metastasis through the Wnt/β-Catenin/Axin2 Pathway. Cancer Res. 2016;76(11):3364-75.

 17       Li X, Liu J, Zhou R, Huang S, Huang S, Chen XM. Gene silencing of MIR22 in acute lymphoblastic leukaemia involves histone modifications independent of promoter DNA methylation. Br J Haematol. 2010;148(1):69-79.

 18       Jung Y, Bang S, Choi K, Kim E, Kim Y, Kim J, et al. TC1 (C8orf4) enhances the Wnt/beta-catenin pathway by relieving antagonistic activity of Chibby. Cancer Res. 2006;66(2):723-8.

 19       Zhu P, Wang Y, Du Y, He L, Huang G, Zhang G, et al. C8orf4 negatively regulates self-renewal of liver cancer stem cells via suppression of NOTCH2 signaling. Nat Commun. 2015;6:7122.

 20       van der Flier LG, Sabates-Bellver J, Oving I, Haegebarth A, De Palo M, Anti M, et al. The Intestinal Wnt/TCF Signature. Gastroenterology. 2007;132(2):628-32.

21       Varnat F, Duquet A, Malerba M, Zbinden M, Mas C, Gervaz P, et al. Human colon cancer epithelial cells harbour active HEDGEHOG-GLI signalling that is essential for tumour growth, recurrence, metastasis and stem cell survival and expansion. EMBO Mol Med. 2009;1(6-7):338-51.

**S1 Table. PCR primers used.**

All primers are listed 5’ to 3’.

**Figure S1. Primary screen for WNT-TCF antagonists and identification of hits**

A) Diagram of the screen set up and dynamic range obtained in the luciferase activity (Firefly/Renilla) readout with various construct combinations as noted. The maximum value obtained by expression of transfected nuclear N’∆βCATENIN (βCAT*) partnering with endogenous TCF factors is equated to 100%. The dynamic range of the screen is set by the difference between this and the value obtained by repression of N’∆βCATENIN activity on the TCF reporter by co-expressed dnTCF, which acts directly on the TCF binding site (TCF-BS). The small molecules tested in the screen are thus expected to repress events in WNT signaling at the level of βCATENIN or below. B) Graphic representation of the top TCF luciferase activity repression hits at or above the value afforded by dnTCF. Those below (examples at right) are considered non-specific or cell toxic. C) List of top 15 natural (top panel) and 14 scaffold (bottom panel) compounds tested with screen ID, TCF-dual luciferase assay results, the normalized expression levels of three TCF targets, two positive (*AXIN2* and *cMYC*) and one negative (*CDKN1A*), and BrdU incorporation. Heat maps show ratios of values for drug treatments over DMSO treatments. No change equals 1 as compared in each case to controls using reporter and N’∆βCATENIN (Luciferase), DMSO (BrdU) and housekeeping genes for *AXIN2*, *cMYC* and *CDKN1A* (p21) for normalization. Highlighted in blue are the 4 compounds selected for further analyses. In red are normalized luciferase (40% or lower) or gene expression levels (for *AXIN2* and *cMYC*, 30% or lower), and in purple, BrdU incorporation results (50% or lower). Green highlights *CDKN1A* levels above 2-fold. D) Chemical structure of the top 4 hits highlighted in blue in (C) with the screen ID and the Analyticon Discovery number of each natural product (NP). E) Heat map of changes afforded by treatment with 10µM for 16h with the top four primary screen hits as compared with changes induced by expression of dnTCF. All numbers are ratios of normalized ct values of drug treatment over DMSO controls. Values at, above or below 30% are highlighted.

**Figure S2. Structures and activities of CAP analogues**

A) Chemical structures of three CAP1 analogues from the Analyticon Discovery libraries with their natural product (NP) number. B) Heat map of the effects of CAP2-4 on an extended WNT-TCF target signature in Ls174T cells after treatment with 10µM for 16h. All values are normalized to controls, which were equated to 1. Values at, above or below 30% are highlighted. For comparison, the results with dnTCF4 and CAP1 from S1E Fig. are shown here. CAP2 and CAP3 were active as CAP1 (in blue boxes) and CAP4 was considered inactive.

**Figure S3.** **Effects of CAP molecules on WNT-TCF reporters, epithelial CC14 colon cancer cell morphology and effects of CAP1, 2 and 3 at 2.5µM.**

A) Histogram of dual TCF luciferase activity results in percentage for 293T cells treated with DMSO carrier control (black bar) or with CAP1-4 at 10µM for 12h. B) CC14 cell behavior after treatment with different concentration of CAP1, CAP2 and CAP3 for 24h as noted. CAP2 leads to the dissolution of typical CC14 epithelial colon cancer islands and eventual death of cells at lower doses than CAP1 or CAP3. CAP1 1µM shows the normal morphology of CC14 cells [see also 21]. C) Inhibitory effects of CAP1-3. Effects of CAP1, 2 and 3 treatments at 2.5µM on relative fluorescence of CC14-GFP^+^ cells over time. Control cells were treated with DMSO, the solvent of CAP1, 2 and 3. See Fig. 1B for differential effects at 0.15µM concentration.

**Figure S4. Effects of cardenolides on WNT-TCF response genes in LS174T cells.**

Heat map of induced changes in gene expression (A) and structures (B) of tested cardenolides. None of the compounds tested (A,B) reproduced the repression of *AXIN2*, *LGR5* and *cMYC*, and the concomitant induction of *CDKN1A* seen by CAP2 or dnTCF4. The clinically approved cardiac glycoside digitoxin only failed to repress *LGR5* (also seen at 100 and 10nM, not shown), indicating that this molecule does not harbor the same activity as CAP2. The heat map (panel A) shows ratios of experimental (0.5µM for 16h) over DMSO-only controls after housekeeping gene normalization. These effects were not directly correlated with the extent of cell death induced after acute treatment (0% cell death with digoxin, gitoxigenin and cymarin, 50% death with digitoxin and 70% death with neriifolin, convallatoxin and oleandrin at the doses used).

**Figure S5. Apoptotic, rescue and general effects of CAP2.**

A) Apoptotic effects of CAP1-3. Increase in the number of cells expressing activated, cleaved Caspase 3 over total live cell numbers (apoptotic index) after treatment with different concentrations of CAP2 as noted. Significant increases in both DLD1 and Ls174T cells appear first at 2.5µM. P values are given as noted in relation to DMSO-treated controls. B) Reversion of CAP2 gene modulation by TCF^VP16^. Heat map of ratios of normalized expression levels of WNT-TCF targets tested for the rescue of the inhibitory effects of CAP2 at 5µM for 16h via expression of TCF^VP16^. The repression of *AXIN2*, *LGR5, cMYC and ASCL2,* as well as the upregulation of *CKN1A,* by CAP2 are reversed by TCF^VP16^ with context-dependent variants. C) Concentration-dependent repression of WNT-TCF targets by CAP2 treatment in different colon cancer cells. The heat map shows ratios over controls of normalized ct values. Multiple cell types are shown to highlight the general action of CAP2.

**Figure S6**. Top microarray changes in gene expression.

Lists of genes as indicated showing upregulation or downregulation by 5-fold of more in the microarray data shown in Fig. 1 after CAP2 or ivermectin treatment compared with the results of Ls174T sibling control cells treated with DMSO for the same time.

**Figure S7. Analysis of transcriptomic changes induced by CAP2 and Ivermectin on human Ls174T colon cancer cells with Panther tools.**

A) Global analyses of up and down changes with CAP2 or Ivermectin from (Fig. 1D) with GO classifier Panther software highlighting molecular function, Protein class and biological processes enriched by drug treatment. The name of the most abundant class for each is given over the correspondent position in the pie charts. The color codes for different partitions are the same for CAP2 and ivermectin. B) Global analysis of the data with the Panther pathway software identifying WNT signaling as the second top hit. The top 14 pathways are represented in the histogram with the y axis given the number of genes included in each category or class. C) Analyses of WNT-TCF, BMP /TGFβ and Cytokine/chemokine pathway hits identified for major components or targets in the microarray data (A). Change is given in fold (positive or negative) and only reliable _at or *_*a_at probes are counted.

**Figure S8. Overlapping effects of selamectin, ivermectin and CAP2 as WNT-TCF response blockers.**

A) Venn diagram of transcriptomic changes observed in microarrays after 5µM selamectin treatment of Ls174T cells for 16h, compared with those induced by similar doses of CAP2 and ivermectin, focusing on the 106 gene signature of dnTCF previously identified [20]. B) List of genes for each category in (A). The common lists, which includes the canonical WNT-TCF targets, is listed in red. C) Selamectin phenocopies CAP2 and ivermectin in the repression of the core 15 TCF target set common to DLD and Ls174T cells [20].

**Figure S9. Okadaic acid sensitivity and gene regulation by CAP2**

A) Okadaic acid sensitivity of the repression of CYCLIN D1 levels by CAP2.

Western blot analyses of the levels of CYCLIN D1 and GAPDH in DLD1 cells treated with DMSO only as control and those treated with 5µM CAP2 +/- 15nM Okadaic acid (OA). GAPDH levels are shown as loading controls. Quantification of CYCLIND1 levels and their normalization by the levels of GAPDH revealed a 2-fold decrease in CAP2 treated cells, which was rescued by okadaic acid co-treatment. B) Heat map showing the comparison of CAP2 responses by Ls174T and DLD1 cells in vitro. Modulation of WNT-TCF, BMP and cytokine gene expression by CAP2 treatment in DLD1 cells much as in Ls174T cells. Values are ratios of experimental (2.5µM CAP2 treatment for 16h) over control (DMSO-only) results after housekeeping normalization.

**Figure S10. WNT-TCF gene expression changes after CAP2 treatment in vivo and in vitro.**

A) Heat map of gene expression changes as noted in dissociated cells from tumors, cultured in vitro and treated with CAP2 at different concentrations. Values above show the results of CAP2 treatment of cells derived from control tumors and those below of cells derived from a tumor previously treated in vivo with CAP2 that recurred. All values were normalized with housekeeping genes and shown as ratios to their respective DMSO-treated controls. Note that all cells are CAP2 responsive. B) Histogram of normalized gene expression levels in dissociated cells after CAP2-treatment as above but equating the level of CAP2 repression in cells derived from DMSO-treated tumors in vivo to 1 (light blue), and comparing this to the level obtained with cells derived from CAP2-treated tumors in vivo, retreated in vitro (darker blue). Note that the levels of WNT-TCF targets is generally lower in cells derived from tumors that were exposed in vivo to CAP2 in comparison to those in cells from tumors that were not treated with CAP2. P values are pairwise as shown.

**Figure S11. Withanolides A and B do not reproduce the effects of CAP2.**

A) Heat maps of normalized rt-qPCR values over control showing that witanolides A, B (0.5µM for 16h) fail to modulate WNT-TCF, cytokine, BMP and epigenetic gene signatures characteristic of CAP2 treatment in Ls174T cells. For comparison, the general heat map result for CAP2 is indicated below, where red means repression and green upregulation.
B) 2D and 3D structures of withanolide F (CAP2), and withanolides A,B from ChemSpider. The 3D structures are aligned through the D ring of the steroid core (middle column) and the steroid core in profile (right column). Note the kink of the lactone of withanolide F in relation to withanolides A and B. In all cases the lactones are to the right.

**Figure S12. Concentration and time effects of CAP2 treatments and regulation of *GLI1*.**

A) Concentration-dependent nanomolar range effects of CAP2.

Modulation of selected genes as in (Fig. 5B) after treatment with 1, 100 and 500nM of CAP2, batch 4, for 16h as indicated. B) *GLI1* regulation by CAP2. Heat map showing ratios of normalized rt-qPCR ct values of CAP2 treatment over DMSO controls for *GLI1*. This key Hedgehog-response gene and mediator is sensitively regulated by the time and dose of CAP2 treatments. CAP2 batch3 was used at 5µM for 2,4 or 16h. CAP2 batch 4 was used at 1, 100 and 500nm for 16h. “–“ refers to the inability to detect *GLI1* in the treated sample by rt.qPCR.
