# Supplementary material for: Long-Lasting WNT-TCF Response Blocking and Epigenetic Modifying Activities of Withanolide F in Human Cancer Cells
Source: PLoS One. 2016 Dec 14;11(12):e0168170. doi: 10.1371/journal.pone.0168170 (PMC5156407; doi:10.1371/journal.pone.0168170)
Supplement: S1 Table — (DOCX) [file pone.0168170.s002.docx]

AXIN2 Fw:      CTCCTTATCGTGTGGGCAGT

AXIN2 Rev:    CCAACTCCAGCTTCAGCTTT

ASCL2 Fw:    GCGTTCCGCCTACTCGT

ASCL2 Rev:  GGCTTCCGGGGCTGAG

LEF1 Fw:       AACCTCTCAGGAGCCCTACC

LEF1 Rev:     CACGGGCACTTTATTTGAT

LGR5 Fw:      GGAGCATTCACTGGCCTTTA

LGR5 Rev:    CTGGACGGGGATTTCTGTTA

cMYC Fw:      TGGTCTTCCCCTACCCTCT

cMYC Rev:     GATCCAGACTCTGACCTTTT

CDKN1A Fw:    GACTCTCAGGGTCGAAAACG

CDKN1A Rev:  AAGATGTAGAGCGGGCCTTT

EFNB1 Fw:    AAGAACCTGGAGCCCGTTC

EFNB1 Rev:  GGGGTCGAGAACTGTGCTAC

EPHB2 Fw:   ATGCGGAAGAGGTGGATGTA

EPHB2 Rev:  CCTTGAAAGTCCCAGATGGA

EPHB3 Fw:   TGCCACTCAAGCTCTACTGC

EPHB3 Rev:  GTTATTGTGGCAGGTGCAGA

HMBS Fw:     AAGTGCGAGCCAAGGACCAG

HMBS Rev:    TTACGAGCAGTGATGCCTACCAAC

PTPRO Fw:   TTCAGAGGAAGAGCAGGACGAC

PTPRO Rev:  CATCCTGCATCTCGTCAGCATA

TBP Fw CCACAGCTCTTCCACTCACA

TBP Rev:    GGATTATATTCGGCGTTTCG

SOX4 Fw:      AAACCAACAATGCCGAGAAC

SOX4 Rev:     GTTCATGGGTCGCTTGATGT

ZNRF3 Fw     CGGGTCATCCCCTGTACTC

ZNRF3 Rev   ACGTGAGAGGTTGCTGGTCT

KITLG Fw:     TTCCATCATAGACAAACTTGTGA

KITLG Rev:    GTAAAGAGCCTGGGTTCTGG

WDR71 Fw:  GTACTGACGGGACCATGAAAA

WDR71  Rev: ATATCTTCAGCTGGGCATCC

C1ORF33 Fw: TGGCAAAAACAAGGTGATGA

C1ORF33 Rev:GAACAGGAGACCCACCTCAC

HSPC111 Fw:CAAGGCCAAGGGCAAAAC

HSPC111 Rev:GGTTCTGCCGTACCGATTTA

ID1 Fw:     GGAATCCGAAGTTGGAACC

ID1 Rev:    GAGACCCACAGAGCACGTAA

CXCL2 FW : CCCTGGCCACTGAACTG

CXCL2 RV : ATGACTTCGGTTTGGGCG

CXCL3 Fw: CGTCCGTGGTCACTGAACT

CXCL3 Rev: ATGACTTCGGTTTGGGCG

IL33 Fw:   TGAGTCTCAACACCCCTCAA

IL33 Rev: GTTGGCATGCAACCAGAAGT

ZIC2 Fw: TTTTTCCGCTATATGCGGCAG

ZIC2 Rev: :TCCACCGAGACGTGTGTCA

HIG2 Fw: TTCTGCGCTGGTGCTTAGTA

HIG2 Rev: GGCTGAAAGGACCCTACTCC

ID3 Fw: ACCTTCCCATCCAGACAGC

ID3 Rev: CTTCCGGCAGGAGAGGTT

NOL1 Fw: TACCATGGGGCGCAAGTT

NOL1 Rev: CTCTTTCGAGCACGACTAGACA

BMP4 Fw: GATCCACAGCACTGGTCTTG

BMP4 Rev: GGGATGCTGCTGAGGTTAAA

GLI1 Fw: AGCGTGAGCCTGAATCTGTG

GLI1 Rev: CAGCATGTACTGGGCTTTGAA

ZMYND8 Fw: CGAGACCCAGAGTAAAGCCAT

ZMYND8 Rv: GATGTATCCGCATAGTCAGGG

EED Fw: GTGACGAGAACAGCAATCCAG

EED Rev: TATCAGGGCGTTCAGTGTTTG

SMARCAL1 Fw: ACAGCATCAGAGGACTAGCTC

SMARCAL1 Rev: CACTGGCTTACAAGACTCCCT

TBL1XR1 Fw: CCAGTTCCCATTTCCTGCCA

TBL1XR1 Rev: ACACCACAAAAGGAGGCACTT

SETD6 Fw: GGAGGGAACAGCAAGCCTTA

SETD6 Rev: TCAGGGAACAGGGAAACTGC

TAF5L Fw: CAGGCAGAACCCCAGCAATA

TAF5L Rev: CACTGTGCTCTTCGGACTGT

β-ACTIN Fw: ACAGAGCCTCGCCTTTGC

β-ACTIN Rev: GGAATCCTTCTGACCCATGC

HPRT Fw: AAGATGGTCAAGGTCGCAAG

HPRT Rev: CTCCAGATGTTTCCAAACTCAAC
