# Supplementary material for: Long-Lasting WNT-TCF Response Blocking and Epigenetic Modifying Activities of Withanolide F in Human Cancer Cells
Source: PLoS One. 2016 Dec 14;11(12):e0168170. doi: 10.1371/journal.pone.0168170 (PMC5156407; doi:10.1371/journal.pone.0168170)
Supplement: S6 Fig — (PDF) [file pone.0168170.s008.pdf]

	CAP2 vs. Control		
	UP		DOWN
HIST1H2BD	52	SYNCRIP	-34.0
ALDH8A1	29	NMI	-32.3
HIST1H2AB /// HIST1H2AE	27	ZMYND8	-28.5
TTC18	25	SLC16A14	-27.8
HIST1H2AG /// HIST1H2AH /// HIST1H2AI /// HIST1H2AK /// HIST1H2AL /// HIST1H2AM	24	VAV3	-27.2
NCALD	23	RFXAP	-24.8
ERVK3-2 /// LOC100506358	23	KIT	-23.8
NPR3	22	SRSF1	-21.5
NOV	21	SPC25	-21.1
CCL4 /// CCL4L1 /// CCL4L2 /// LOC101060278	20	PPIL1	-20.7
SERPINC1	19	ARMCX5	-20.0
EGR1	19	**PTPRO**	-19.3
HIST2H2BE	18	ELK3	-18.6
HIST1H2BC /// HIST1H2BE /// HIST1H2BF /// HIST1H2BG /// HIST1H2BI /// NCALD	18	PCK1	-18.1
SLC17A8	15	SH3PXD2A	-18.0
HIST2H2AA3 /// HIST2H2AA4	15	CXCL3	-17.9
HIST1H1C	14	FAIM	-17.9
SSR1	14	KLHL23 /// PHOSPHO2-KLHL23	-17.8
MDM2	13	GJA1	-17.3
HIST1H2BC /// HIST1H2BE /// HIST1H2BF /// HIST1H2BG /// HIST1H2BI	13	DCAF16	-17.2
OSGIN1	13	GCNT3	-16.9
FBXO11	12	ITPRIPL2	-16.3
CDKL2	12	ADORA2B	-16.2
LTB4R	12	MLKL	-15.7
GLS	12	HNF4G	-15.5
ADRBK2	11	PLEKHA2	-15.5
CYP2C9	11	STEAP1	-15.5
PLGLA /// PLGLB1 /// PLGLB2	11	TRAF5	-15.3
GUCA1B	11	SORBS2	-15.2
[illegible]1H4D /// HIST1H4E /// HIST1H4F /// HIST1H4H /// HIST1H4I /// HIST1H4J /// HIST1H4K /// HIST1H4L /// HIST2H4A /// HIST2H4B /// HIST4H4	11	**LGR5**	-15.1
WDR96	10	POLE2	-15.1
LSMEM1	10	PRMT6	-14.3
TNFSF13B	10	KBTBD11	-14.3
TICAM2 /// TMED7-TICAM2	10	PPP1R9A	-14.2
ARRDC4	10	PDGFC	-14.1
ATF3	10	QSER1	-14.0
SRXN1	10	FUT3	-13.9
SCG5	10	ZBTB38	-13.8
TDRD6	9	MYB	-13.6
CD200R1	9	MAGEF1	-13.6
ACTG1P4 /// AMY1A /// AMY1B /// AMY1C /// AMY2A /// AMY2B	9	AIM1	-13.5
MSH4	9	KIAA0226L	-13.5
SCARNA14	9	RABGAP1L	-13.0
SOX30	9	UTRN	-13.0
ACTC1	9	DIXDC1	-13.0
PRDM2	9	SCAMP1	-12.8
CDKN1A	9	EFNA4	-12.8
MYRFL	8	IL33	-12.7
DHRS2	8	ZNF165	-12.5
ITGAM	8	TM4SF1	-12.5
ITGA9	8	**PTGDR**	-12.4
RNF150	8	SPIN4	-12.2
LRRC8C	8	SMARCAD1	-12.1
CHRNA10	8	TRMT11	-12.0
HIST1H2BK	7	SP100	-11.8
HIST1H2BJ	7	PHLDA1	-11.7
HIST1H2AC	7	SLITRK6	-11.7
CORIN	7	POLR3G	-11.6
RASA4 /// RASA4B	7	CXCL1	-11.5
LAT2	7	IRF2BP2	-11.5
LDHAL6B	7	ANKRD22	-11.4
HIST1H3A /// HIST1H3B /// HIST1H3C /// HIST1H3D /// HIST1H3E /// HIST1H3F /// HIST1H3G /// HIST1H3H /// HIST1H3I /// HIST1H3J	7	N4BP2L2	-11.4
PLAGL1	7	KCNN4	-11.3
MBP	7	PAIP1	-11.3
ALLC	7	TUBBP5	-11.3
BTG2	7	ZCCHC10	-11.3
CCDC11	7	LGALS8	-11.3
ZNF211	7	CASD1	-11.3
PYGM	7	EMG1	-11.2
RHOB	7	C2CD2	-11.2
SERTAD1	7	USP46	-11.1
NFYA	6	TRABD2A	-11.1
ERRFI1	6	WDFY3	-11.0
TXK	6	CMPK2	-10.9
SRP19 /// ZRSR1	6	STEAP2	-10.9
KIAA1683	6	RIMBP2	-10.9
TLL2	6	ACBD5	-10.9
CLEC4A	6	CXCL2	-10.8
C12orf5	6	WT1	-10.7
MGEA5	6	KIAA1430	-10.6
SCARNA20	6	INTS6	-10.6
PDZK1 /// PDZK1P1	6	EREG	-10.6
LOC101060503 /// TXNIP	6	SH3BP2	-10.5
MIR22 /// MIR22HG	6	VPS54	-10.4
CHST7	6	AGR3	-10.4
LOC100507577 /// LONP2	6	SLC35G1	-10.3
POPDC2	6	CLASP2	-10.3
C18orf25	6	DMRTA2	-10.2
MOBP	6	CDX1	-10.2
SERPINE1	6	UNC50	-10.2
CREBRF	6	MYC	-10.2
TP53INP1	6	TSEN2	-10.2
STX16	5	UPRT	-10.1
ACTG1P4 /// AMY2A /// AMY2B	5	PRR15L	-10.1
HIST1H2AK	5	KLHL13	-10.1
LRRC39	5	FAM169A	-10.1
DNHD1	5	ZNF827	-9.9
FOXD4 /// FOXD4L1 /// FOXD4L3 /// FOXD4L6 /// LOC101060560	5	E2F8	-9.9
CRYGS	5	ZNF532	-9.9
CDKN2D	5	ELOVL6	-9.9
SECISBP2	5	TNFSF10	-9.9
ZNF791	5	SSTR1	-9.8
TGOLN2	5	UHRF1BP1	-9.8
DCAF4L1	5	RTN4IP1	-9.8
OSBPL8	5	LXN	-9.8
KLF6	5	FUNDC1	-9.7
TARDBP	5	DENND2D	-9.7
LEFTY1	5	ZHX2	-9.7
HIPK1	5	DLG3	-9.7
		STEAP1 /// STEAP1B	-9.7

NFIA	-9.6
PSMG4	-9.5
ADSS	-9.5
NSMCE4A	-9.5
DEPDC1	-9.5
MYADM	-9.4
TGIF2	-9.4
NDC1	-9.4
EPHX4	-9.3
SLC9A2	-9.3
CRLS1	-9.3
SLC38A1	-9.2
LGALSL	-9.2
KCTD20	-9.2
MTPAP	-9.2
CFL2	-9.2
METAP2	-9.2
MSH2	-9.2
CAPRIN1	-9.2
TACC2	-9.2
MAP3K5	-9.2
AGPS	-9.2
GPATCH2	-9.1
SQLE	-9.1
METTL21A	-9.1
ASCL2	-9.1
FASTKD2	-9.0
RAD51AP1	-9.0
PBDC1	-9.0
C4orf19	-9.0
TMEM68	-9.0
PRKD3	-8.9
ERBB2IP	-8.8
USP24	-8.8
TRIM2	-8.8
SRSF7	-8.8
RNF125	-8.7
UTP18	-8.7
C2CD4A	-8.7
SCLT1	-8.7
FUBP1	-8.7
FNBP1L	-8.6
PRKCH	-8.6
TJP2	-8.6
ZNF518A	-8.6
SNTB1	-8.5
NUDT12	-8.5
TRMT61B	-8.5
NAV2	-8.5
TOX	-8.5
MET	-8.5
RANBP2	-8.5
BRWD1	-8.5
KIN	-8.5
IPO8	-8.4
MDFIC	-8.4
MIR17 /// MIR17HG /// MIR18A /// MIR19A /// MIR19B1 /// MIR20A /// MIR92A1	-8.3
MANSC1	-8.3
PLA2G3	-8.3
SRSF3	-8.3
LNX1	-8.3
C8orf44-SGK3 /// SGK3	-8.3
GTF2H2 /// GTF2H2B /// GTF2H2C /// GTF2H2D	-8.2
DNAH14	-8.2
ZDHHC23	-8.2
FGFBP1	-8.2
ARHGAP32	-8.2
STAMBPL1	-8.2
MOB1B	-8.1
CAND1	-8.1
AGO4	-8.1
LOC100190940	-8.1
ABHD13	-8.0
CD58	-8.0
OR51E1	-8.0
ARMC8	-8.0
DTWD1	-8.0
RRM2	-8.0
NCAPG	-8.0
UBE2J1	-8.0
LHFPL2	-8.0
BIVM	-8.0
MTHFS /// ST20-MTHFS	-8.0
C19orf12	-8.0
ATP2B1	-7.8
ZC3HAV1L	-7.8
C8orf4	-7.8
METTL8	-7.8
SREK1IP1	-7.8
FAM105A	-7.8
STXBP6	-7.7
GTF2IRD1	-7.7
CDC14B	-7.7
PLA2G4A	-7.7
MYCL1	-7.7
OVOL2	-7.6
ZNF681	-7.6
PLSCR1	-7.5
SLC43A3	-7.5
EED	-7.5
NHLRC3	-7.5
BAG2	-7.5
NAA15	-7.5
GIMAP2	-7.5
UBE2Q2	-7.5
XRCC4	-7.5
ID4	-7.5
RPS6KB1	-7.5
SASS6	-7.4
DDX52	-7.4
RBM12	-7.4
MRPL15	-7.4
IFIH1	-7.4
BUB1B	-7.4
PLEKHA5	-7.4

MYO1B	-7.4
BCL11B	-7.4
TCEAL8	-7.3
LARP7	-7.3
BRCA1	-7.3
TMPO	-7.3
CKAP4	-7.3
CHML	-7.3
TOMM40L	-7.3
NAPEPLD	-7.3
WDR36	-7.3
KIF11	-7.3
PHF2	-7.2
GPR126	-7.2
SKIV2L2	-7.2
HIVEP1	-7.2
ZFHX3	-7.2
MMACHC	-7.2
FAM13A	-7.1
ANGEL2	-7.1
MACC1	-7.1
TCEAL1	-7.1
EVA1A	-7.1
PTPRB	-7.1
EXOG	-7.1
SMAD6	-7.1
CASP1	-7.1
NAMPT	-7.1
GPR180	-7.1
XKRX	-7.0
SEH1L	-7.0
RAP2C	-7.0
PSMG1	-7.0
ZMYM2	-7.0
KCTD14 /// NDUFC2-KCTD14	-7.0
IQGAP2	-7.0
PEX3	-7.0
USP13	-7.0
TET3	-6.9
TIFA	-6.9
SPIN2A /// SPIN2B	-6.9
SLC4A7	-6.9
THUMPD2	-6.9
HNRNPDL	-6.9
MKI67	-6.9
MT4	-6.8
GTPBP8	-6.8
ESRP1	-6.8
IL20RA	-6.8
TMEM216	-6.8
CEBPD	-6.8
ZNF689	-6.8
PTGS2	-6.8
KRIT1	-6.8
PCDHB2	-6.7
PNN	-6.7
DCLRE1A	-6.7
RAB28	-6.7
PPP6C	-6.7
OPA1	-6.7
CAV2	-6.7
ADAT2	-6.7
PARP9	-6.7
HNRNPU	-6.7
TTF2	-6.6
SIMC1	-6.6
BCL2	-6.6
GINS3	-6.6
PSME4	-6.6
EEF1E1	-6.6
MBTPS2	-6.6
PRLR	-6.6
ZNF260	-6.6
SP8	-6.6
FUT4	-6.6
TLK1	-6.5
ZNF639	-6.5
CFLAR	-6.5
C15orf52	-6.5
DSC2	-6.4
ZNF248	-6.4
HUNK	-6.4
C9orf64	-6.4
PWP1	-6.4
OAS1	-6.4
PATZ1	-6.4
CSTF3	-6.4
FDXACB1	-6.4
CELF2	-6.4
PTPN3	-6.4
LIPG	-6.4
CLDN1	-6.3
KIAA1199	-6.3
DEPTOR	-6.3
HMGA2	-6.3
PPP2R1B	-6.3
ABHD12B /// MIR4454	-6.3
MAGOHB	-6.3
SLC25A43	-6.3
ITGB8	-6.3
CRADD	-6.3
TMEM30B	-6.3
RPS14	-6.3
ZBED1	-6.3
TMEM168	-6.3
RWDD3 /// TMEM56-RWDD3	-6.3
ZNF618	-6.3
PHACTR2	-6.2
BUB3	-6.2
IGF2BP3	-6.2
AKAP1	-6.2
ATP1B1	-6.2
ANKRD13C	-6.2
QTRTD1	-6.2
MAML3	-6.2

VEZT	-6.2
ERICH1	-6.1
GHR	-6.1
ITGA6	-6.1
GLRX	-6.1
SMARCAL1	-6.1
UCHL5	-6.1
PTGER4	-6.1
TMEM246	-6.1
SHANK2	-6.1
SLC17A5	-6.1
METTL1	-6.1
GPR39	-6.1
LOC100509484 /// PLIN2	-6.1
PDS5B	-6.1
KLHL7	-6.0
TMEM56	-6.0
TNS3	-6.0
NUP35	-6.0
FUT2	-6.0
SNX9	-6.0
NASP	-6.0
TCERG1	-6.0
PCGF6	-6.0
ENPP4	-6.0
DENND4C	-6.0
ZNF711	-6.0
SENP7	-6.0
KNSTRN	-6.0
CFTR	-6.0
PDE5A	-5.9
NUDCD1	-5.9
PRDM8	-5.9
CLPX	-5.9
RPAIN	-5.9
XK	-5.9
CD24	-5.9
INPP1	-5.9
C9orf40	-5.9
VASH2	-5.9
IRS1	-5.9
GJB2	-5.9
ATP7B	-5.9
PRDM16	-5.8
ZNF32	-5.8
NIF3L1	-5.8
NF1	-5.8
LYSMD2	-5.8
KLHL42	-5.8
ZMYM6 /// ZMYM6NB	-5.8
ZNF189	-5.8
ELF2	-5.8
NFIC	-5.8
MND1	-5.8
E2F5	-5.7
PARP2	-5.7
MTMR1	-5.7
MPZL2	-5.7
MCPH1	-5.7
HEATR1	-5.7
TBL1XR1	-5.7
SETD6	-5.7
ZNF232	-5.7
RQCD1	-5.6
FYCO1	-5.6
CCDC58	-5.6
TRIM32	-5.6
MDGA2	-5.6
C1orf106	-5.6
PLCB4	-5.6
RFC3	-5.6
MKX	-5.6
IVNS1ABP	-5.6
CAMK2N1	-5.6
ZBED6CL	-5.6
SBF2	-5.6
TAF5L	-5.6
THRA	-5.6
LEPROTL1	-5.6
SMIM3	-5.6
AP4M1	-5.6
SEMA3A	-5.6
CHRAC1	-5.5
ASXL1	-5.5
SRSF8	-5.5
TMEM117	-5.5
BEND3	-5.5
TLR3	-5.5
PCDHB14	-5.5
IL8	-5.5
TRAPPC13	-5.5
FNDC3A	-5.5
ID2	-5.5
EEPD1	-5.4
KIAA1586	-5.4
SLC11A2	-5.4
SLTM	-5.4
BET1	-5.4
LDOC1L	-5.4
HOMER1	-5.4
LTV1	-5.4
NR2C1	-5.4
CDC7	-5.4
MCM3	-5.4
TAMM41	-5.4
RAB27A	-5.4
PPM1A	-5.4
NUP98	-5.4
DTX3L	-5.4
IRAK1BP1	-5.3
IMPACT	-5.3
DDX60	-5.3
MON2	-5.3
KIAA0391	-5.3
YAP1	-5.3

ERLEC1	-5.3
GGA2	-5.3
ISCA1	-5.3
ARID2	-5.3
GNAI1	-5.3
CTPS2	-5.3
TBC1D9	-5.3
ZW10	-5.3
TOX3	-5.3
ZFYVE16	-5.3
SLC37A1	-5.3
MRPS31	-5.3
SMPDL3A	-5.3
GPRC5A	-5.3
COX20	-5.3
TRAK2	-5.2
AJUBA	-5.2
BBX	-5.2
RNF2	-5.2
PIK3AP1	-5.2
PVRL3	-5.2
TM7SF3	-5.2
SUV420H1	-5.2
POLA2	-5.2
SH3RF3	-5.2
TBC1D8B	-5.2
HNRNPD	-5.2
TMEM39A	-5.2
SAP30	-5.2
DENND5B	-5.2
DIMT1	-5.2
RNF145	-5.2
CSRP2BP /// PET117	-5.2
NUP88	-5.2
SH3RF2	-5.2
NEDD4	-5.2
RPGR	-5.2
XIAP	-5.2
STAU2	-5.2
TRIB2	-5.2
YARS2	-5.2
NFKB1	-5.2
IFT81	-5.2
TIPIN	-5.2
RIF1	-5.2
ICK	-5.2
DOCK9	-5.1
IFT74	-5.1
FAR2	-5.1
ANKRD32	-5.1
RNF138	-5.1
AKAP2 /// PALM2-AKAP2	-5.1
RNF144A	-5.1
SRP19	-5.1
RAB11FIP2	-5.1
PPP4R1	-5.1
ROCK2	-5.1
ZNF641	-5.1
LYPLAL1	-5.1
SENP6	-5.1
TMEM218	-5.1
UNC93A	-5.1
LURAP1L	-5.0
UTP20	-5.0
KIF21A	-5.0
QRSL1	-5.0
MYEOV	-5.0
GNPTAB	-5.0
IBTK	-5.0
LPPR1	-5.0
AGFG1	-5.0
PRKAA1	-5.0
ZNF321P /// ZNF816 /// ZNF816-ZNF321P	-5.0
ZNF608	-5.0
CCDC50	-5.0
SNX13	-5.0

Ivermectin vs. Control

	UP		DOWN
CASP5	37.8	*MT4*	-24.6
RAB39B	28.1	*CXCL5*	-21.5
ATF3	24.9	*FGFBP1*	-16.1
DDIT3	24.8	*UGT2B15*	-16.0
BEX2	24.5	*HMGB3*	-15.5
ISG20	21.4	***PTGDR***	-15.1
IFIT1	18.5	***PTPRO***	-14.1
OASL	16.7	***OLFM4***	-13.4
IRF9	16.6	*SMAD6*	-10.8
PEAR1	16.4	*C10orf99*	-10.3
IL1A	15.9	*OR51E1*	-9.4
CDKN2B	12.4	*ID1*	-8.6
EGR1	11.1	*INSIG1*	-8.4
FILIP1L	10.6	***EPHB3***	-8.4
GADD45A	10.2	*ID4*	-8.3
TSLP	9.8	*PLA2G3*	-8.1
C8orf4	9.3	*CYB5B*	-7.6
H1F0	9.3	*CCNE2*	-7.0
IFIT2	8.9	*MIR636 /// SRSF2*	-6.8
ANXA1	8.9	*FAM198B*	-6.7
IFI44	8.6	*SLC39A10*	-6.5
FAM129A	8.6	*FABP1 /// PRDM10*	-6.5
VIMP	8.4	*KIAA0101*	-6.4
SLC22A15	8.0	*SMOC2*	-6.3
ZFAND2A	7.9	*H3F3A /// H3F3B /// MIR4738*	-6.1
HKDC1	7.9	*CCNE1*	-6.0
WIPI1	7.7	***LGR5***	-6.0
ZNF222	7.6	*MCM3*	-6.0
SPINK1	7.6	*HMGB1*	-5.9
CLGN	7.4	*HNRNPU*	-5.9
AREG /// AREGB	7.3	*ADRA2A*	-5.6
FBXO16	7.2	*ETS2*	-5.6
XDH	6.8	*C2CD4A*	-5.5
C6orf223	6.8	*ISX*	-5.4
HSPA13	6.8	*ATP5G3*	-5.4
FGF21	6.4	*SRSF11*	-5.3
GDF15	6.3	*DEFA6*	-5.3
SERPINB8	6.3	*HSPA1A /// HSPA1B*	-5.3
MIR22 /// MIR22HG	6.3	*PDZD2*	-5.3
ARMCX3	6.3	*MYBL1*	-5.2
GTPBP2	6.2	*ID2*	-5.2
NFIL3	6.2	*RASL11A*	-5.1
CREB5	6.0	*GINS2*	-5.1
PECR	6.0	*MYB*	-5.1
PTPRH	5.9	*FAM111B*	-5.0
ZCCHC2	5.9	*METTL7A*	-5.0
LRRC2	5.8	*CYTIP*	-5.0
TRIB3	5.8	*LYZ*	-5.0
SEPP1	5.8		
INHBE	5.8		
MYH4	5.7		
SLC30A1	5.7		
DAPP1	5.7		
CREB5 /// LOC401317	5.6		
LAMP3	5.5		
SELM	5.4		
DNAJB9	5.4		
CALCA	5.4		
ARHGAP29	5.3		
FOS	5.2		
TMEM39A	5.2		
SQSTM1	5.1		
LYPD6B	5.1		
FKBP14	5.1		
IRF7	5.1		
GK	5.1		
CHD2 /// LOC100507217	5.1		
TMEM154	5.1		
GADD45G	5.0		
RWDD2A	5.0		
CYP39A1	5.0		
